# The importance of temperature fluctuations in understanding mosquito population dynamics and malaria risk

**DOI:** 10.1098/rsos.160969

**Published:** 2017-03-08

**Authors:** Lindsay M. Beck-Johnson, William A. Nelson, Krijn P. Paaijmans, Andrew F. Read, Matthew B. Thomas, Ottar N. Bjørnstad

**Affiliations:** 1 Department of Biology, Center for Infectious Disease Dynamics, The Pennsylvania State University, University Park, PA, USA; 2 Department of Entomology, Center for Infectious Disease Dynamics, The Pennsylvania State University, University Park, PA, USA; 3 Department of Biology, Queen’s University, Kingston, Ontario, Canada; 4 ISGlobal, Barcelona Ctr. Int. Health Res. (CRESIB), Hospital Clinic, Universitat de Barcelona, Barcelona, Spain; 5 Fogarty International Center, National Institutes of Health, Bethesda, MD, USA

**Keywords:** temperature fluctuation, mosquito population dynamics, age structure, malaria risk, seasonality, delay-differential equations

## Abstract

Temperature is a key environmental driver of *Anopheles* mosquito population dynamics; understanding its central role is important for these malaria vectors. Mosquito population responses to temperature fluctuations, though important across the life history, are poorly understood at a population level. We used stage-structured, temperature-dependent delay-differential equations to conduct a detailed exploration of the impacts of diurnal and annual temperature fluctuations on mosquito population dynamics. The model allows exploration of temperature-driven temporal changes in adult age structure, giving insights into the population’s capacity to vector malaria parasites. Because of temperature-dependent shifts in age structure, the abundance of potentially infectious mosquitoes varies temporally, and does not necessarily mirror the dynamics of the total adult population. In addition to conducting the first comprehensive theoretical exploration of fluctuating temperatures on mosquito population dynamics, we analysed observed temperatures at four locations in Africa covering a range of environmental conditions. We found both temperature and precipitation are needed to explain the observed malaria season in these locations, enhancing our understanding of the drivers of malaria seasonality and how temporal disease risk may shift in response to temperature changes. This approach, tracking both mosquito abundance and age structure, may be a powerful tool for understanding current and future malaria risk.

## 1. Introduction

Environmental conditions have the potential to alter disease transmission and intensity, a fact frequently seen in vector-borne diseases. In most vector-borne disease systems, the vector is a small ectotherm that is sensitive to the ambient environmental conditions, such as temperature. Arguably, the most devastating vector–pathogen combination for humans today is *Plasmodium* parasites that cause malaria and the *Anopheles* mosquitoes that transmit them. Malaria represents a large burden on global public health, and while great strides have been made over recent years in controlling this disease, it is still a major problem for about half of the world population [1]. The influence that temperature and fluctuations in temperature have on the risk of malaria transmission is not well characterized at the population level [2], though the importance of fluctuations has been well characterized for individual life-history traits [3–5]. Gaining a better understanding of the impacts of temperature on vector dynamics and malaria transmission will increase our ability to control malaria both now and into the future.

*Anopheles* mosquitoes are sensitive to temperature throughout their life cycle, with development, birth and mortality rates all dependent on temperature [4–11]. The *Anopheles* mosquito life cycle consists of three aquatic juvenile stages (egg, larva and pupa) and adult. Adult female *Anopheles* mosquitoes take blood meals from vertebrate hosts every few days, depending on temperature, for egg development [10]. It is through this cyclical blood-feeding process that the *Plasmodium* parasites can be picked up from infected hosts and subsequently transmitted to susceptible hosts. A critical feature of *Plasmodium* life history is the extrinsic incubation period (EIP), the developmental period inside the mosquito before the parasite becomes transmissible [12–15]. The length of the EIP is dependent on temperature, generally taking 8–14 days to develop, so most adult *Anopheles* mosquitoes are likely to die before the age at which they could potentially transmit the parasites [16–18]. Thus, age structure is a key determinant of the population’s capacity to vector malaria parasites [4,17,19–21]. Populations with longer-lived adults (relative to EIP) produce more effective vectors because more adults reach the epidemiologically relevant age. The relationships with temperature differ between life-history traits (e.g. mortality versus development) and mosquito life stages (e.g. larva versus adult), leading to complex, and non-intuitive population level responses to temperature [22]. Optimal temperatures for larval development are not necessarily optimal for survival; similarly, the optimum temperature for reproduction and adult survival may differ from that of the parasite development rate. Because of these nonlinearities, impacts of temperature on population dynamics and vector competence are complex [22].

Most empirical and theoretical work has focused on the impacts of constant or mean monthly temperature on various aspects of mosquito or parasite life histories [5,22–24], though recently some studies have focused on more realistic temperature variation [25]. Temperature variation has been shown, both theoretically and empirically, to be important in mosquito-borne disease systems, including malaria, and leads to dynamics that differ from those gathered at constant temperatures [2–5,25]. However, these studies have mostly focused on individual life-history traits, and have not explored the interactions and complexities between traits to create a complete picture of the impact of temperature fluctuations on transmission. Exploring population-level responses to temperature fluctuations will lead to better understandings of population dynamics in the field and may generate more effective intervention strategies. Additionally, a comprehensive exploration of the effects of different magnitudes and frequencies, annual or diurnal, of variability, will allow for the identification of important amplifiers of malaria risk.

Temperature, but also rainfall, humidity and land-use, are known important drivers of mosquito dynamics and malaria risk [26–31]. Each of these, in combination with anthropogenic factors such as intervention strategies, determines the dynamics of local mosquito populations and the intensity of malaria transmission. These factors are complex and may be of greater or lesser importance depending on location. Here, we first focus on temperature and the effects of seasonal and diurnal fluctuations on mosquito population dynamics and parasite EIP, and thus the potential for malaria transmission. We then explore the combined effects of temperature and precipitation in determining the malaria season at four locations in Africa.

## 2. Methods

The model used to explore the effects of temperature fluctuations on mosquito population dynamics is a stage-structured, delay-differential equation (DDE) model from Beck-Johnson *et al.* [22]; it reflects our current understanding of temperature-dependence in the mosquito life cycle, and for convenience is detailed in the electronic supplementary material. The model stages mirror the main stages in the mosquito life cycle, with temperature-dependent developmental time lags that vary in length depending on current conditions. Birth and mortality rates are also temperature-dependent. Laboratory studies on the effects of temperature on various life-history rates [7–10] were used to parametrize the model. The model assumes that density-dependence manifests in larval stage mortality rate, that the baseline mortality rate for the larval stage is temperature-dependent, and that mortality resulting from density-dependence is additive.

In addition to tracking abundance of larval and adult stages, we also track abundance of adult mosquitoes that have lived long enough to be potentially infectious with malaria. A mosquito can, at the earliest, become infected with malaria when it takes its first blood meal; however, in our model, we make the simplifying assumption that mosquitoes can be exposed to *Plasmodium* as they enter the adult stage. This method does not explicitly model malaria dynamics, rather using the predicted adult survivorship and temperature-dependent EIP of the parasite, we can calculate the abundance of adults that can potentially be infectious.

The temperature-dependent EIP function is

2.1 $$\mathrm{EIP}=D\left(\exp\left(-{\left(\frac{T(t)}{L_{2}}\right)}^{K_{2}}\right)-\exp\left(-{\left(\frac{T(t)}{L_{1}}\right)}^{K_{1}}-{\left(\frac{T(t)}{L_{2}}\right)}^{K_{2}}\right)\right),$$

where *T*(*t*) is temperature at time *t* and *D*,*L*_*i*_ and *K*_*i*_ (*i*=1 or 2) are scalars. This function reflects that the EIP starts to lengthen at very warm temperatures which has been shown to be important (electronic supplementary material, figure S1) [4,32]. The function is similar in shape to that proposed by Paaijmans *et al.* [4], but continuous and bounded by zero, making it more tractable for dynamic models.

We explored model dynamics using annual, diurnal, and combined annual and diurnal sinusoidal temperature fluctuations. We investigate annual fluctuations with four different annual temperature ranges of 4°C, 8°C, 12°C and 16°C, chosen to broadly reflect the annual ranges at different latitudes across malaria-endemic regions in sub-Saharan Africa [33–35]. The effect of diurnal fluctuation was investigated with two temperature ranges of 8°C and 14°C to explore the differences between small and large daily temperature ranges, respectively. Finally, we investigate the combined effects of annual and diurnal temperature fluctuations. We explore each for mean temperatures ranging from 16°C to 40°C, covering the range of temperatures that are important for the malaria system [22]. All models were integrated numerically using the package PBSddesolve in R (R v. 2.15.3) [36,37]; the code is available as electronic supplementary material.

Our model gives temperature-based predictions per litre of larval habitat, which allows us to scale our results by the availability of larval habitat. While the relationship between *Anopheles* abundance and larval habitat or precipitation is not directly predictable, there is a clear positive relationship between them [23,28,38,39]. Because the amount of rainfall converted into larval habitat is a highly complex process involving many local factors [27,40,41], we cannot make a blanket prediction about dynamics of larval habitat in response to precipitation. However, following previous studies, we assume that monthly precipitation less than 80 mm is insufficient to sustain malaria transmission [23,42]. We used this threshold precipitation level to explore the interaction between precipitation, temperature and predicted malaria risk.

To explore this interaction we chose four locations that are within the malaria transmission zone in sub-Saharan Africa: Birao, Central African Republic; Libreville, Gabon; Victoria Falls, Zimbabwe and Xai-Xai, Mozambique (electronic supplementary material, figure S2). These were chosen because daily temperatures and precipitation were available for an extended period of time and they represent a range of environmental conditions (electronic supplementary material, table S1). We ran the model with recorded daily mean temperatures from an historic period ranging from 16 to 19 years depending on data availability and completeness, as follows: Birao, 1961–1978, Libreville, 1960–1977, Victoria Falls, 1969–1985 and Xai-Xai, 1961–1980. Model results were summarized as mean annual potentially infectious mosquito populations and were filtered by the precipitation threshold. Mosquitoes were tracked from the egg stage; any eggs laid in months where precipitation was above the threshold were assumed to be in the population that could eventually become disease vectors. We assumed mosquitoes could no longer enter the population when precipitation dropped below the threshold, but mosquitoes already in the population were tracked until mortality removed them. Note that precipitation data were not included in model simulations, rather it is used to determine when mosquito populations would be present given the temperature-dependent model predictions. Because the precipitation threshold is a monthly measure, daily precipitation data were combined by month. The data are freely available from NOAA, National Climatic Data Center [43]. The mosquito temperature-dependent development and mortality functions were assumed to be the same at all for locations. This assumption does not allow for exploration of variation between populations but is reasonable given the lack of data available for parametrizing mosquito life-history parameters (for a full discussion of model parametrization, see Beck-Johnson *et al.* [22]).

At each location, the historic malaria season was established from the MARA/ARMA project [23,44], which provides malaria season months for Africa, and confirmed by estimates of months of risk and observed cases [45–47]. We examined the ability of precipitation and temperature-driven mosquito abundance to explain the malaria season using generalized linear models (GLM) with binomial errors. These models were ranked with Akaike information criterion with a finite sample size correction (AICc). We used these historic malaria season data deliberately as they enabled us to explore the effects of environmental conditions on fundamental dynamics of malaria without confounding issues of large-scale adoption of interventions such as long-lasting insecticidal nets and indoor residual sprays that have shaped transmission considerably in recent times. However, for Xai-Xai, data are available for the period from 1980 to 2000, and results from this time period were compared with the results from the 1961–1980 period. The temperature data from 1990 to 2000 is much less complete than the data before 1990 [43] so the results focus on the ten year period from 1980 to 1990.

## 3. Results

### 3.1. Sinusoidal temperature forcing

To explore in detail the dynamic consequences of variable temperatures, we explored a subset of four mean temperatures (18, 22, 26, 30°C) across the fourteen different fluctuating temperature regimes (see electronic supplementary material for a full description of the results for all temperature fluctuations and mean temperatures explored). We found that mosquito populations were more likely to have sustained year-round populations when mean temperatures were 22 or 26°C and were more likely to go through a seasonal crash with a mean temperature of 18 or 30°C (electronic supplementary material, figures S3–S5). With a mean temperature of 30°C, the model predicted population extinctions more frequently than with the other three temperatures, particularly when the temperature fluctuation was large.

**Figure 1:**
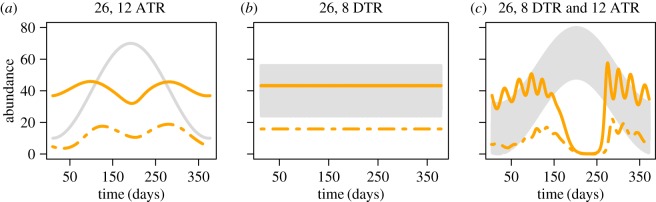
Annual cycles of abundance from one mean temperature (26°C) with three different seasonal drivers. Annual cycles of adult mosquito abundance (orange solid lines), abundance of potentially infectious mosquitoes (dashed orange lines) and the temperature fluctuation (grey line or band). The abundance is number of mosquitoes per litre of larval habitat. (*a*) 12°C annual temperature range, (*b*) 8°C diurnal temperature range and (*c*) 8°C diurnal temperature range nested within a 12°C annual temperature range.

Examining predicted population abundances across the temperature fluctuations at a single mean temperature, we found that the size and type of fluctuation can greatly alter the predicted population outcome (figure 1 and electronic supplementary material, S3–S5). To illustrate this, we focused on three of the fluctuating temperature drivers with a mean temperature of 26°C. We found that adult mosquito abundance was predicted to have a very slight seasonal dip with the 12°C annual temperature range, to be constant over time with the 8° diurnal temperature range, and to go through a seasonal crash when those two drivers were combined (figure 1). The predicted abundance of potentially infectious mosquitoes showed similar differences between temperature fluctuations. This result was even more notable as the size differences between the compared fluctuations increased or when the mean temperature was closer to the edge of the temperature range explored.

**Figure 2:**
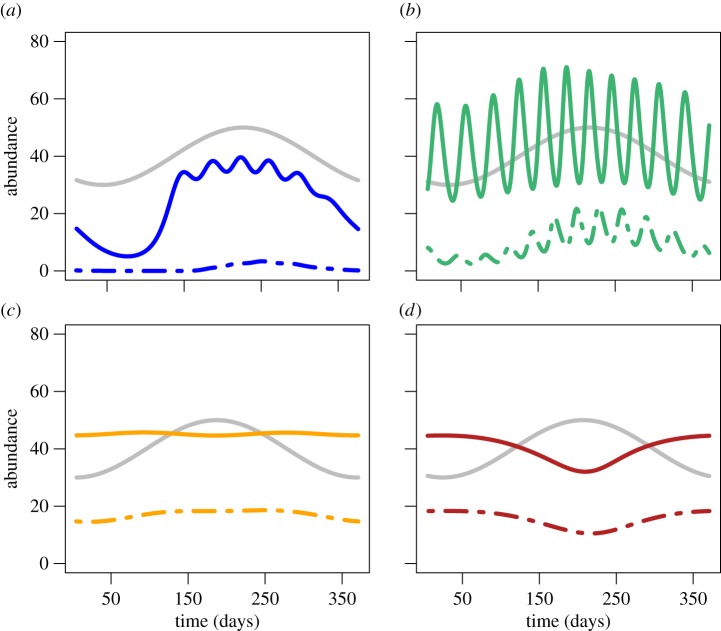
Annual cycles of abundance from four mean temperatures with the same seasonal driver (4°C annual temperature range). Annual cycles of adult mosquito abundance (solid lines), abundance of potentially infectious mosquitoes (dashed lines) and the temperature fluctuation (grey lines). The abundance is number of mosquitoes per litre of larval habitat. (*a*) Mean of 18°C, (*b*) mean of 22°C, (*c*) mean of 26°C *and* (*d*) mean of 30°C.

The abundance of potentially infectious adults was found to be influenced by both mean temperature and fluctuation (figure 2 and electronic supplementary material, figures S3–S5). However, the dynamics of this age group did not have a consistent relationship with those of adult mosquitoes. To illustrate this, we focused on the results from the four mean temperatures mentioned above (18, 22, 26, 30°C), with a 4°C annual temperature range. We found that the relationship between the dynamics of adults and potentially infectious adults differed across mean temperatures (figure 2). At mean temperatures of 18 and 22°C adult population dynamics poorly approximated the dynamics of the potentially infectious adults, which showed a greater amount of seasonality and, in the case of 18°C, was non-existent for most of the year (figure 2*a*,*b*). When mean temperature was 26°C, the adult population was a decent predictor of the potentially infectious abundance; however, the latter shows a very slight seasonal cycle that the adult abundance did not show (figure 2*c*). When mean temperature was 30°C, we found that the dynamics of the adults and the potentially infectious adults matched (figure 2*d*). The relationships between mean temperature and predictability of potentially infectious mosquitoes from the adult abundance did not hold in other temperature scenarios.

**Figure 3:**
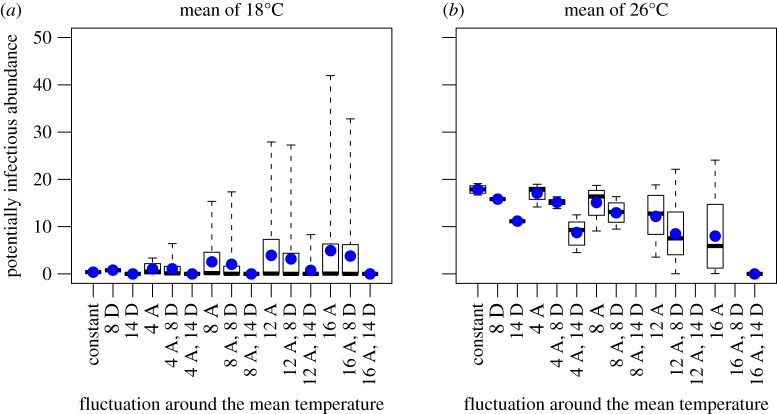
Comparison of the mean, median and variation across temperature fluctuation types and sizes. Panels (*a*) and (*b*) show the abundance of the potentially infectious adult population (number of mosquitoes per litre of larval habitat) predicted by the model driven by both the constant and all the fluctuating temperature drivers. Panel (*a*) shows results for a mean temperature of 18°C and panel (*b*) shows results for a mean temperature of 26°C. The *x*-axis is the temperature driver, where constant denotes constant temperature, D denotes a diurnal fluctuation and A denotes an annual fluctuation. The numbers along the *x*-axis (4, 8, 12 and 14) indicate the size of the temperature fluctuation around the mean temperature. For example, 4A, 8D refers to a 4°C annual and an 8°C diurnal fluctuation. The *x*-axis is arranged in order of increasing annual fluctuation. The box and whiskers show the total variation and the median for each fluctuation and the blue dots show the mean abundance.

We found the mean total adult abundance predicted from the fluctuating drivers to be consistently lower than the mean predicted at constant temperature (constant temperature results in [22]; electronic supplementary material, figure S6). In contrast, at low mean temperatures, the mean potentially infectious adult abundance with fluctuating temperatures was predicted to be higher than the mean abundance predicted at constant temperature (figure 3). In other words, fluctuations around low mean temperatures can increase the potentially infectious population even though they decreased the overall adult population. At warmer temperatures, fluctuations decreased the potentially infectious population mean (figure 3), thus temperature fluctuations may either increase or decrease malaria risk.

The population cycle magnitude of adults and potentially infectious adults increased as annual temperature fluctuation increased in comparison with the constant temperature results at all mean temperatures explored (figure 3 and electronic supplementary material, S6). Warmer mean temperatures damped the population cycles compared with cooler mean temperatures, suggesting that populations at cooler temperatures are more prone to temperature-driven boom and bust cycles than are populations at warmer temperatures. Diurnal temperature fluctuations tended to decrease the magnitude of population cycles in comparison with the annual temperature driver alone; the larger the diurnal temperature driver, the larger the decrease in population cycle. This was not true at all mean temperatures; for example, at a mean temperature of 18°C the superimposition of an 8°C diurnal fluctuation on a 4°C annual temperature fluctuation increased the population cycles in both adult and potentially infectious adult populations. This detailed exploration of the range in abundances agrees with the general trend at all 25 mean temperatures across the 14 fluctuating conditions (electronic supplementary material, figures S7–S13).

### 3.2. Real-world temperature forcing

**Figure 4:**
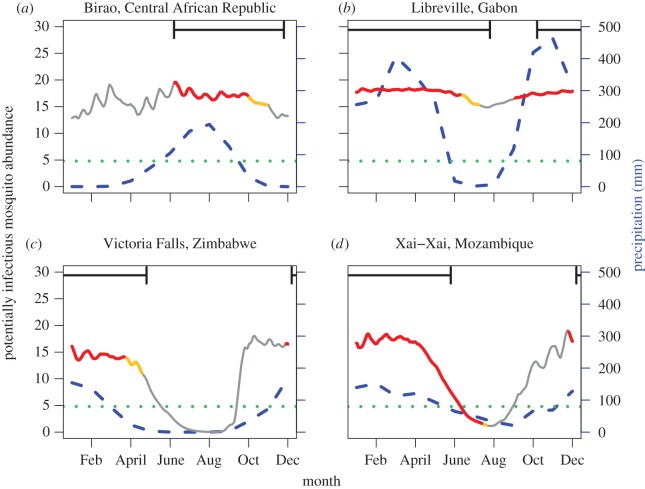
Mean annual mosquito population dynamics, filtered by water availability. These plots show the model predictions for the potentially infectious mosquito population. The left *y*-axis corresponds to the mosquito population abundance (number of mosquitoes per litre of larval habitat) and the right *y*-axis corresponds with precipitation. The blue dashed line is the mean monthly precipitation and the horizontal green dotted line shows the 80 mm precipitation threshold. The mosquito population line has been colour coded to correspond with the availability of water. Recall that the model is not directly run with precipitation information, but is rather filtered once the temperature-dependent results are established. The red portions of the line correspond to populations in which the eggs were laid when precipitation was equal to or above the 80 mm per month threshold. The orange portions of the mosquito population line shows the populations where precipitation has fallen below the threshold but mosquitoes that were already in the potentially infectious class are surviving. The greyed out portion of the line shows what the population would do if water was available year round. The black horizontal time index bars at the top of the panels mark the beginning and end of the malaria season. The panels are (*a*) Birao; (*b*) Libreville; (*c*) Victoria Falls and (*d*) Xai-Xai.

To fully explore the interactions between temperature and precipitation on predicted malaria risk and to check our model’s ability to correctly forecast seasonal malaria risk, we used observed daily mean temperatures from the four locations in sub-Saharan Africa to drive our model. Birao and Libreville were predicted to have fairly stable potentially infectious mosquito populations year-round; this is particularly true of Libreville. In contrast, Victoria Falls and Xai-Xai were predicted to have seasonal drops in potentially infectious mosquito populations. The results of these simulations (see raw simulation results in electronic supplementary material, figure S14) were then filtered by the observed precipitation. At all locations, the combination of the temperature-driven mosquito population abundance from our model and the observed precipitation data predicted the observed malaria season better than precipitation or temperature alone (figure 4 and electronic supplementary material, table S2).

At three of the focal locations, Birao, Libreville and Victoria Falls, there is a delay between the increase in precipitation above the 80 mm threshold and the onset of the malaria season and a similar delay between the decrease in precipitation and the end of the malaria season (figure 4*a*–*c*). The time lag before the start of the malaria season can be explained by the temperature-dependent development time in the mosquito population. Additionally, the delay between the fall in precipitation and the end of the season can be explained by the temperature-dependent survival of potentially infectious mosquitoes (orange portion of line figure 4). For example, the four- to six-month malaria season in Birao [23,44,45], ends a month after precipitation falls below the 80 mm threshold. No mosquitoes are added to the population after the precipitation falls below the threshold; however, the survival of mosquitoes already in the population accounts for about half the lag time between the decrease in precipitation and the end of the malaria season.

Xai-Xai shows a slightly different interaction between precipitation and temperature than the other three locations explored. At Xai-Xai, the beginning of the malaria season is driven by precipitation. However, at the end of the malaria season there is marked decline in predicted potentially infectious mosquito abundance because of reduced temperature suitability which begins before precipitation drops below the threshold. Additionally, the survival of mosquitoes already in the potentially infectious stage is predicted to be very short. We also explored the dynamics of Xai-Xai in the more recent time period (1980–1990). The predicted mosquito population dynamics are similar to those predicted when the model was run with older temperature data with the same seasonality (electronic supplementary material, figures S15 and S16). The amount of precipitation the site receives was the most marked difference between the two time periods. Because of this, the months which are predicted to have enough precipitation to sustain transmission based on the threshold are reduced from six to five months.

## 4. Discussion

Temperature is an important yet complex driver of mosquito population dynamics. Temperature fluctuations have been shown to have profound impacts on predictions of mosquito and malaria parasite development time [4,5]. Mosquitoes are sensitive to temperature throughout their life cycle and, because temperature dependencies are nonlinear and differ between life-history traits and life stages, it is difficult to predict the population level responses to temperature fluctuations. Using a stage-structured, temperature-dependent DDE model, we performed a thorough exploration of the impacts of temperature fluctuations on mosquito population dynamics and risk of malaria transmission. Our results indicate that fluctuation magnitude and type can greatly change the predicted population dynamics around any mean temperature. Our results show that the dynamics of mosquitoes that have lived long enough to be potentially infectious with malaria parasites does not necessarily mirror those of adult dynamics, suggesting that variable temperatures can alter the age structure of the adult population through time in a biologically significant fashion. The changes to age structure through time and the decoupling of the age structure dynamics from overall adult dynamics would not be captured by a mean temperature driver.

Temperature fluctuations have been shown by empirical and theoretical work to be important in determining the speed of individual life-history traits of mosquitoes and malaria parasites [3,4,42,48]. Our model provides the first exploration of the interactions between temperature-driven life-history traits throughout the mosquito life cycle and parasite development. This has further demonstrated the importance of temperature fluctuations on mosquito population dynamics as a whole. When driven with the same mean temperature but different temperature fluctuations, our model predicts dramatically different population outcomes. The range of mean temperatures where populations are predicted to persist without crashing shifts based on the fluctuation. Fluctuation type and size also determine the presence of overcompensation and annual cycles. There are temperatures at which the adult population dynamics are not predicted to shift much, except in the case of very large fluctuations (e.g. 26°C). However, potentially infectious adult abundance does not necessarily follow the same pattern.

Age structure in *Anopheles* populations is an important factor in the ability of a given population to transmit malaria parasites [4,19,20]; populations with long-lived individuals (relative to the EIP) are better at transmitting parasites than short-lived ones. Little is known, however, about how population age structure changes temporally, or in response to environmental conditions. Our model provides an opportunity to understand how temperature, and fluctuations thereof, may alter age structure and transmission dynamics. The model predicts that in some cases adult abundance is fairly constant through time, but that potentially infectious mosquito abundance is seasonal, further suggesting that the two groups do not necessarily act in lock-step. Additionally, simulations with mean temperatures at the edges of the explored temperature range suggest that the presence of adult mosquitoes does not always result in mosquitoes surviving long enough to be potential vectors (figure 1*a*). These observations lend support to the idea that knowing the age structure of adults in addition to abundance may be important in predicting malaria risk. This finding has implications for other diseases where the age structure of the population determines transmission risk, particularly other mosquito-borne diseases including dengue, Japanese encephalitis virus and West Nile virus [3,49,50], which have relatively long EIPs. Age-grading wild caught mosquitoes is difficult and can be time consuming and expensive [19,51]. Given the importance of mosquito age structure to malaria risk, further development and testing of new age-grading techniques under field conditions would be beneficial for understanding vector populations and ground-truthing our models. Additionally, there is evidence that age-dependent mortality may be important for understanding disease risk [52,53]; it would therefore be useful to explore the effects of age-dependent mortality on temporal variation in age structure.

Many environmental factors influence malaria, including precipitation [23,26–31]. To understand how interactions between fluctuating temperature and precipitation govern seasonality, we used an established precipitation threshold [23,42] to gauge how our temperature-derived predictions of malaria risk change with precipitation availability. While this approach does not address the absolute quantity of larval habitat available, or the scaling of the model-predicted mosquito abundance, it does provide a standardized way of ascertaining whether the population will reach abundances that can sustain malaria transmission. This study does not take into account the biological variation in responses to temperature that may be present in mosquito populations at these four locations. While this variation may be important, the data to parametrize models to population-specific levels is lacking (for a full discussion of model parametrization, see Beck-Johnson *et al.* [22]). The results show that temperature and precipitation influence the duration of the malaria season and that there are location-specific differences in the interaction between the two drivers. This result could help monitor and predict malaria seasonality shifts in response to changing climate. At all locations explored, there is a delay between increasing precipitation and the start of the malaria season while the mosquito population increases. A similar delay occurs when precipitation falls below the threshold and the mosquito population dies off before the end of the malaria season, except at Xai-Xai where reducing temperature suitability results in a rapidly declining mosquito population before precipitation limitations take effect.

Although the sample of locations explored in this study is limited, the differing temperature and precipitation profiles demonstrate this model can be used over a range of environmental conditions and locations. The temperature and precipitation data used here, are from time periods before long-lasting insecticidal nets and indoor residual sprays were implemented widely. These data are more robust that more recent records and were also from a time before the malaria season was confounded by mass interventions, allowing us to explore the mosquito population without confounding issues of population control measures. However, the changing climate makes exploring more recent data important for identifying changes that may occur in seasonality. The comparison between the model results from temperature data from different time periods at Xai-Xai showed only slight changes. At this location the most notable difference was in the precipitation data which showed a decrease in the months where precipitation was above the threshold. Further changes to precipitation and temperature are likely to lead to changes in the predicted malaria season. Future work in this area can extend these analyses to a more comprehensive study of changing seasonality patterns in malaria-endemic regions.

Incorporating temperature into our understanding of mosquito populations and the potential for malaria transmission has already been shown to alter the predicted results about the optimal temperatures for malaria transmission [22,32]. It has also been established that temperature fluctuations can be important when predicting the rates of life-history traits for both the malaria parasite and the mosquito [4,5,42]. Here we show that temperature fluctuations can impact the predicted population dynamics of mosquitoes. Given the same mean temperature, population dynamics vary greatly between the different fluctuating temperature drivers. This work has also demonstrated that in the face of temperature variability the age structure of adult mosquitoes does not remain constant but rather fluctuates over time. Surprisingly, the dynamics of mosquitoes old enough to be potentially infectious can be somewhat dissociated from total adult abundance, suggesting that different nonlinearities in *Plasmodium* and mosquito development induce complex temperature-dependence in malaria risk. The temporal variation in age structure and the disconnect from total adult abundance does not happen under mean temperature conditions. Understanding the driving environmental forces behind *Anopheles* population dynamics is crucial for understanding aspects of malaria transmission. Many vector-borne diseases have long extrinsic incubation periods, so it is possible that the shifts in age structure described here will be important in other disease systems. Intervention strategies and control efforts could be substantially assisted by using information about vector population dynamics. This model with the ability to track both total abundance and age structure represents an innovative tool for furthering these efforts.

## Supplementary Material

Supplementary Methods and Results for Beck-Johnson et al. 2016

## Supplementary Material

Code for Running Mosquito Population Model Simulations (Beck-Johnson et al. 2016)

## Supplementary Material

seasonality.csv

## Data accessibility

The code for simulating the DDE model is included in the supplemental information. The data to perform the GLM analysis has been included in the electronic supplementary material. Additionally, the climatic data for the four African locations are freely available through the NOAA National Climatic Data Center [43].
