# Supplementary material for: The importance of temperature fluctuations in understanding mosquito population dynamics and malaria risk
Source: R Soc Open Sci. 2017 Mar 8;4(3):160969. doi: 10.1098/rsos.160969 (PMC5383843; doi:10.1098/rsos.160969)
Supplement: Supplementary Methods and Results for Beck-Johnson et al. 2016 [file rsos160969supp1.pdf]

Supplementary material for

The importance of temperature fluctuations in understanding mosquito population dynamics and malaria risk

Lindsay M. Beck-Johnson[1,*], William A. Nelson[2], Krijn P. Paaijmans[3], Andrew F. Read[1,4,5], Matthew B. Thomas[4], Ottar N. Bjørnstad[1,4,5]

[1]Department of Biology, Center for Infectious Disease Dynamics, The Pennsylvania State University, University Park, PA, USA;
[2]Department of Biology, Queen's University, Kingston, Ontario, Canada;
[3]ISGlobal, Barcelona Ctr. Int. Health Res. (CRESIB), Hospital Clinic, Universitat de Barcelona;
[4]Department of Entomology, Center for Infectious Disease Dynamics, The Pennsylvania State University, University Park, PA, USA;
[5]Fogarty International Center, National Institutes of Health, Bethesda, MD, USA;
[*]L.Beck-Johnson@colostate.edu;
[*]Present address: Lindsay M. Beck-Johnson, Department of Biology, Colorado State University, Fort Collins, CO, USA

Supplemental Methods

The equations for the temperature-dependent stage-structured model are shown below for convenience. A full description of the model, assumptions and parameters can be found in Beck-Johnson *et al.* 2013 [22].

The state equations are (egg ($E(t)$), larva ($L(t)$), pupa ($P(t)$), adult ($A(t)$)):

$$\frac{dE(t)}{dt} = R_E(t) - R_L(t) - \delta_E(t)E(t) \quad (1)$$

$$\frac{dL(t)}{dt} = R_L(t) - R_P(t) - \delta_L(t)L(t) \quad (2)$$

$$\frac{dP(t)}{dt} = R_P(t) - R_A(t) - \delta_P(t)P(t) \quad (3)$$

$$\frac{dA(t)}{dt} = R_A(t) - \delta_A(t)A(t) \quad (4)$$

where $R_i(t)$ ($i = E, L, P$, or A) is the recruitment into or out of a state and $\delta_i(t)$ represents the per capita, stage-specific mortality rate. The recruitment into a stage is dependent on the recruitment into the previous stage according to,

$$R_E(t) = b(t)A(t) \tag{5}$$

$$R_L(t) = R_E(t-\tau_E(t))S_E(t)\frac{h_E(t)}{h_E(t-\tau_E(t))} \tag{6}$$

$$R_P(t) = R_L(t-\tau_L(t))S_L(t)\frac{h_L(t)}{h_L(t-\tau_L(t))} \tag{7}$$

$$R_A(t) = R_P(t-\tau_P(t))S_P(t)\frac{h_P(t)}{h_P(t-\tau_P(t))} \tag{8}$$

where, $\tau_i(t)$ is the duration of stage i at time t. The egg-laying rate (the number of eggs per female per day) is given by $b(t)$ and the temperature-dependent, stage specific development rate is $h_i(t)$. The length of the delay, $\tau_i(t)$, is determined by the temperature-dependent development rate $h_i(t)$ of stage i at time t.

$S_i(t)$ represents the survival through stage i and expands as follows,

$$S_E(t) = \exp\left(-\int_{t-\tau_E(t)}^{t} \delta_E(\xi)d\xi\right) \tag{9}$$

$$S_L(t) = \exp\left(-\int_{t-\tau_L(t)}^{t} \delta_L(\xi)d\xi\right) \tag{10}$$

$$S_P(t) = \exp\left(-\int_{t-\tau_P(t)}^{t} \delta_P(\xi)d\xi\right) \tag{11}$$

$$S_A(t) = \exp\left(-\int_{t-\tau_A(t)}^{t} \delta_A(\xi)d\xi\right). \tag{12}$$

The stage-specific per capita mortality rate equations ($\delta_i(t)$) are given by,

$$\delta_E(t) = \gamma_E\mu_3\exp\left(\left(\frac{T(t)-\mu_4}{\mu_5}\right)^2\right) \tag{13}$$

$$\delta_L(t) = \gamma_L\mu_3\exp\left(\left(\frac{T(t)-\mu_4}{\mu_5}\right)^2+\sigma L(t)\right) \tag{14}$$

$$\delta_P(t) = \gamma_P\mu_3\exp\left(\left(\frac{T(t)-\mu_4}{\mu_5}\right)^2\right) \tag{15}$$

$$\delta_A(t) = \mu_0\exp\left(\left(\frac{T(t)-\mu_1}{\mu_2}\right)^4\right) \tag{16}$$

where μ_j ($j = 0,1,2,3,4,$ or 5) is a scalar and γ_i ($i = E,L,$ or P) is the proportion of the juvenile life cycle that the eggs, larvae and pupae take up, respectively. The extra mortality experienced by

the larvae because of density-dependence is denoted by σ.

Supplemental Results

The amplitude of the predicted seasonal mosquito population cycle increased as the magnitude of the annual temperature range increased with each of the four annual fluctuations (4, 8, 12, 16°C), around mean temperatures ranging from 16–40°C (Figure S7 & S8). This pattern was most distinct at the edges of the temperature range, with large amplitude cycles populating the middle range of temperatures only as the seasonality increased. Mean temperatures in the middle of the temperature range experienced smaller amplitude cycles because the fluctuations shifted the model over parameter space where mortality is low (e.g. Figures 1 & 2); as the mean temperature shifted away from the middle of the range, fluctuations moved the parameter space between higher and lower mortality rates, creating large cycles (e.g. Figure 2A). The cycle length corresponded to the annual temperature driver with which we forced the model. Population abundances at some of the temperatures, particularly those in the middle of the tested range, displayed overcompensation cycles caused by density-dependence.

Low amplitude overcompensation cycles in the larval population were predicted, which were damped or absent in the adult population when driving the model with either the 8 or 14°C diurnal fluctuation, around mean temperatures ranging from 16 to 40°C. These overcompensation cycles did not occur when mean temperatures were between 22 and 24°C for the 8°C diurnal fluctuation (Figure S9). This type of low amplitude overcompensation cycle at certain temperatures was discussed in our previous study [22]. The amplitude of the overcompensation cycles was much lower than the amplitude of cycles resulting from temperature fluctuations and will likely be overshadowed in the field by other population perturbations. We will therefore focus on the cycles resulting from temperature variability. The population abundance in general decreased when moving from smaller to larger diurnal temperature ranges. The range of mean temperatures at which mosquito populations are predicted to persist was reduced for both the diurnal temperature range explored when compared to the annual temperature range outputs.

A combination of the results from the separate annual and diurnal runs were produced when the model was driven with each combination of the annual and diurnal temperature ranges (Figures S10–S13). Annual fluctuations occurred, increasing in amplitude as the annual temperature range increased. The temperatures at which populations are predicted to persist corresponded to those seen in runs with only the diurnal driver when the annual temperature ranges were 4°or 8°C. When the annual temperature ranges were 12°or 16°C, the range of temperatures at which populations are predicted to persist was contracted when compared to the diurnal driver. This was seen most dramatically when the largest diurnal range (14°C) was combined with the largest annual temperature range (16°C) (Figure S13).

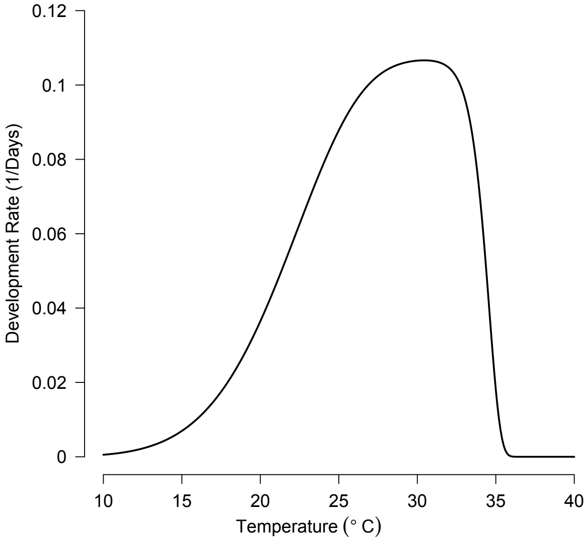

Figure S1: **Temperature dependent malaria parasite development rate.** The temperature-dependent parasite development curve used to predict the length of the extrinsic incubation period in our model. The x-axis is temperature in Celsius and the y-axis is the development rate in 1/days.

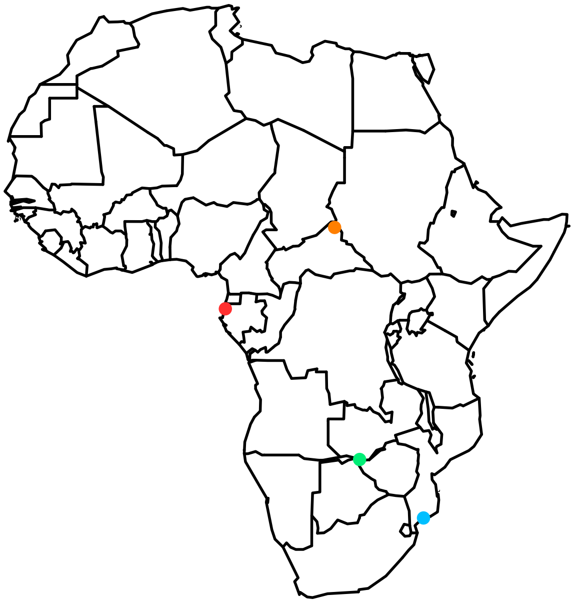

Figure S2: **Locations of precipitation filtering case studies** Orange dot: Birao, Central African Republic. Red dot: Libreville, Gabon. Green dot: Victoria Falls, Zimbabwe. Blue dot: Xai-Xai, Mozambique.

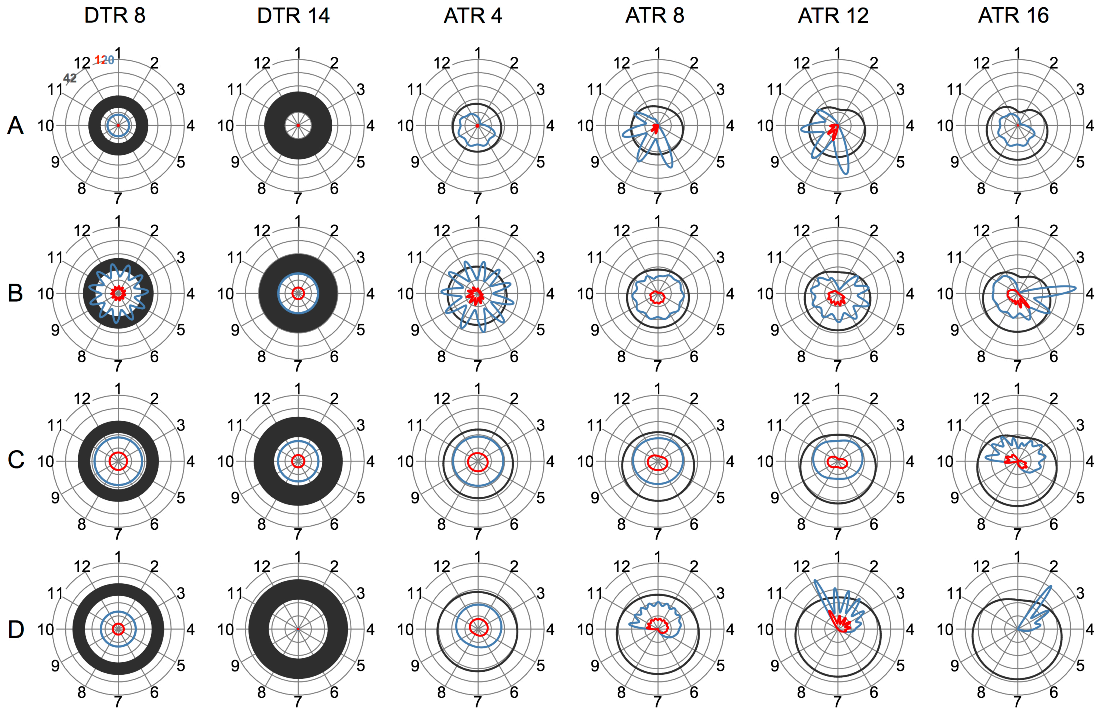

Figure S3: **Annual population dynamics with seasonal or diurnal fluctuation.** Temperature function (dark gray line or band) and the equilibrium abundance of adults (blue line) and potentially infectious mosquitoes (red line) across the two diurnal drivers and the four annual drivers. The abundance is number of mosquitoes per liter of larval habitat. The columns going from left to right are 8, 14°C diurnal temperature range and 4, 8, 12, and 16°C annual temperature range. The rows correspond to the mean temperature with A) 18°C B) 22°C C) 26°C and D) 30°C. The radians correspond with months of the year, going clockwise from 1 to 12. Note the axis scale is different for the temperature function and the mosquito population lines are different. The top left plot shoes the upper limit of the axes for the three lines in the corresponding color; they all start at 0, the temperature function plot goes to 42°C and the two mosquito lines go to 120. The axes are the same for all the radial plots.

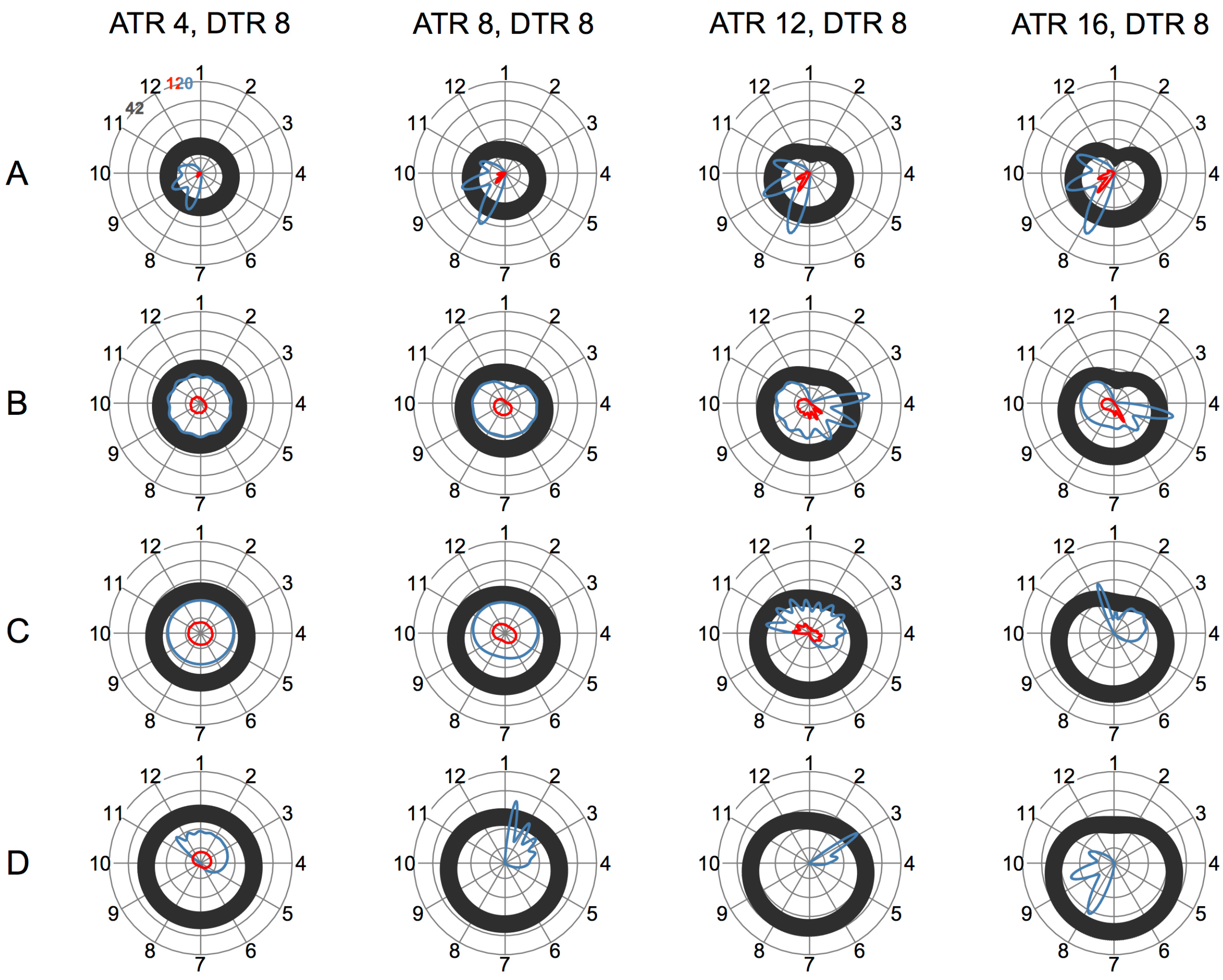

Figure S4: **Annual population dynamics with seasonal and diurnal fluctuation.** Temperature function (dark gray band) and the equilibrium abundance of adults (blue line) and potentially infectious mosquitoes (red line) across the combinations of the four seasonal drivers and the 8°C diurnal driver. The abundance is number of mosquitoes per liter of larval habitat. The columns, going from left to right, have an 8°C diurnal temperature range combined with a 4, 8, 12 or 16°C annual temperature range. The rows correspond to the mean temperature with A) 18°C B) 22°C C) 26°C and D) 30°C. The radians correspond with months of the year, going clockwise from 1 to 12. Note the axis scale is different for the temperature function and the mosquito population lines are different. The top left plot shoes the upper limit of the axes for the three lines in the corresponding color; they all start at 0, the temperature function plot goes to 42°C and the two mosquito lines go to 120. The axes are the same for all the radial plots.

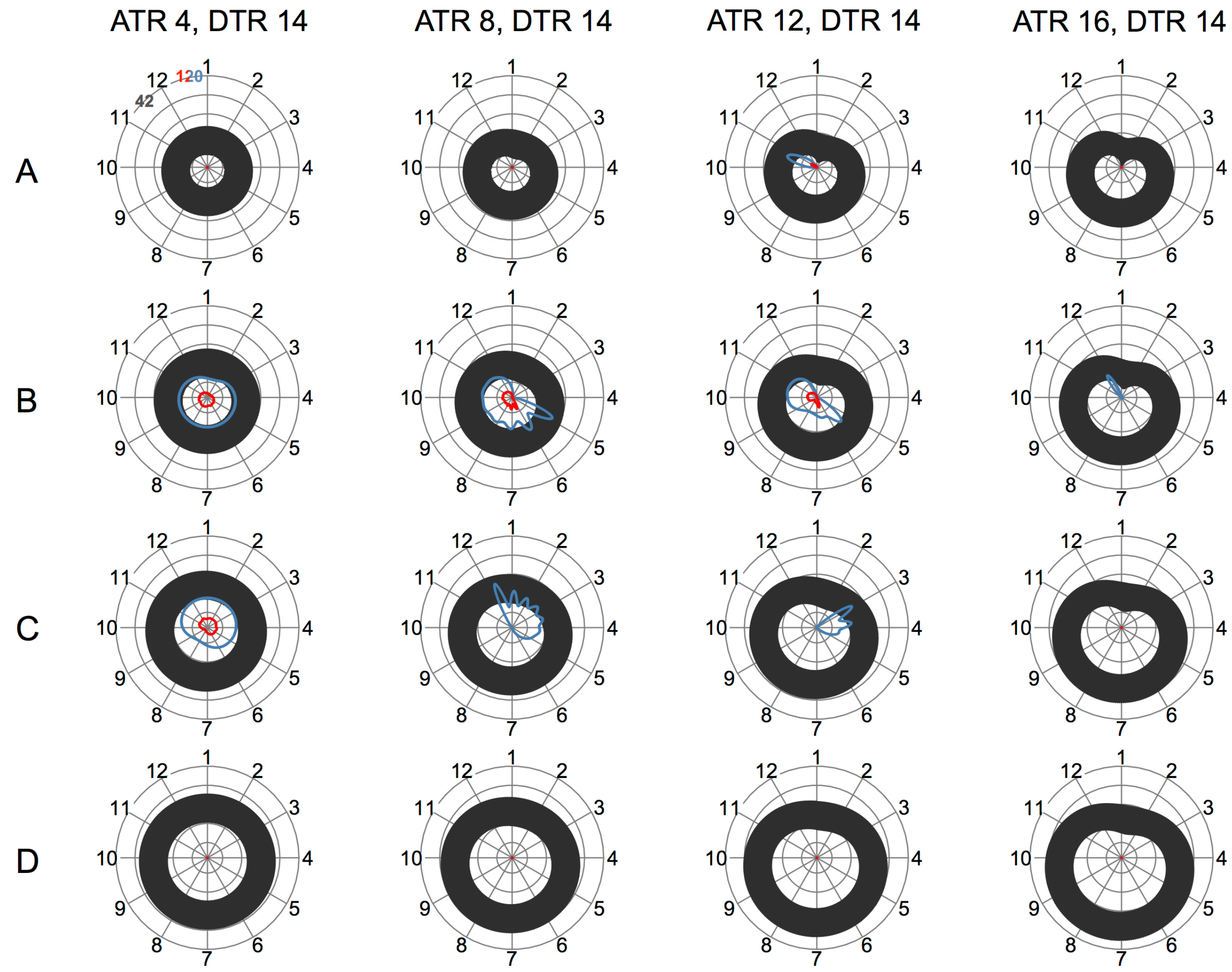

Figure S5: **Annual population dynamics with seasonal and diurnal fluctuation.** Temperature function (dark gray band) and the equilibrium abundance of adults (blue line) and potentially infectious mosquitoes (red line) across the combinations of the four seasonal drivers and 14°C diurnal driver. The abundance is number of mosquitoes per liter of larval habitat. The columns, going from left to right, have a 14°C diurnal temperature range combined with a 4, 8, 12 or 16°C annual temperature range. The rows correspond to the mean temperature with A) 18°C B) 22°C C) 26°C and D) 30°C. The radians correspond with months of the year, going clockwise from 1 to 12. Note the axis scale is different for the temperature function and the mosquito population lines are different. The top left plot shoes the upper limit of the axes for the three lines in the corresponding color; they all start at 0, the temperature function plot goes to 42°C and the two mosquito lines go to 120. The axes are the same for all the radial plots.

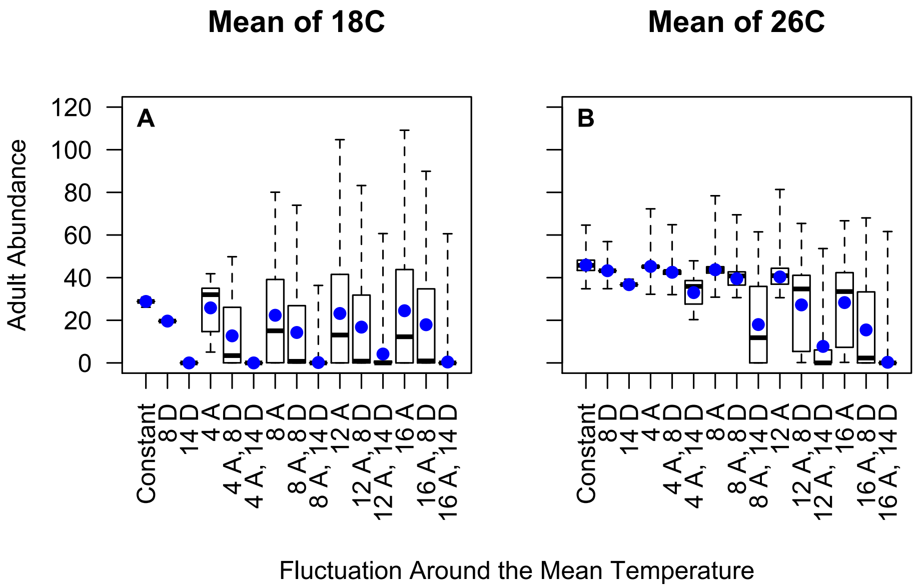

Figure S6: **Comparison of the mean, median and variation across temperature fluctuation types and sizes.** Panels A) and B) show the abundance (number of mosquitoes per liter of larval habitat) of the adult population predicted by the model driven by both the constant and all the fluctuating temperature drivers. Panel A) shows results from a mean temperature of 18°C and panel B) shows results from a mean temperature of 26°C. The x-axis is the temperature driver, with Constant denoting constant temperature, D denoting a diurnal fluctuation and A denoting an annual fluctuation. The x-axis is arranged in order of increasing annual fluctuation. The box and whiskers show the total variation and the median for each fluctuation and the blue dots show the mean abundance.

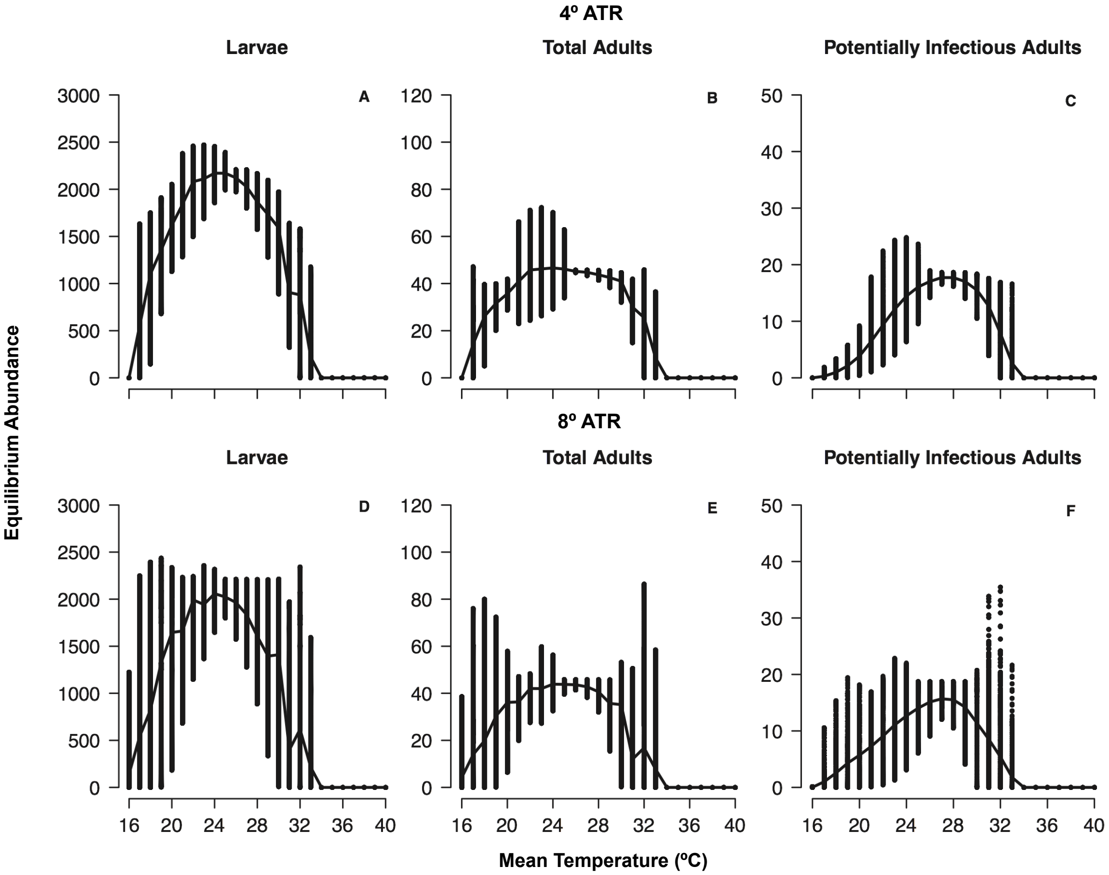

Figure S7: **Equilibrium abundance of model runs using a annual temperature driver.** The x-axis is the mean temperature around which the temperature is fluctuated. The vertical bars are the abundances (number of mosquitoes per liter of larval habitat) that the system cycles through at equilibrium. The line going through the bars is the average abundance. Panel A, B and C are the larval, total adult and potentially infectious adult abundance with a 4°C annual temperature range, panel D, E and F are the larval, total adult and potentially infectious adult abundance with an 8°C annual temperature range.

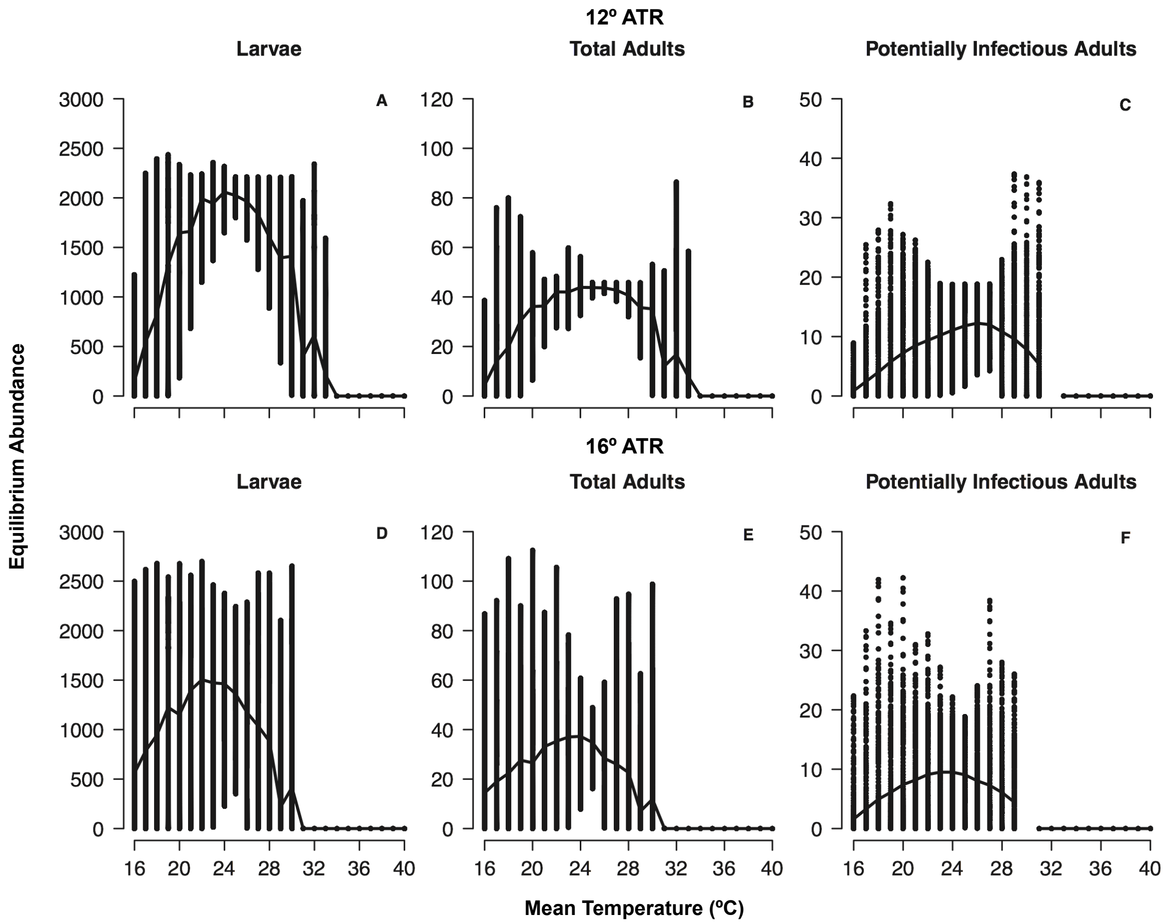

Figure S8: **Equilibrium abundance of model runs using a annual temperature driver.** The x-axis is the mean temperature around which the temperature is fluctuated. The vertical bars are the abundances (number of mosquitoes per liter of larval habitat) that the system cycles through at equilibrium. The line going through the bars is the average abundance. Panel A, B, and C are the larval, total adult and potentially infectious adult abundance with a 12°C annual temperature range, panel D, E, and F are the larval, total adult and potentially infectious adult abundance with a 16°C annual temperature range. At some combinations of high temperature and large temperature fluctuations the solver was too stiff to handle the rapid changes in temperature and could not accurately capture the dynamics of the potentially infectious population.

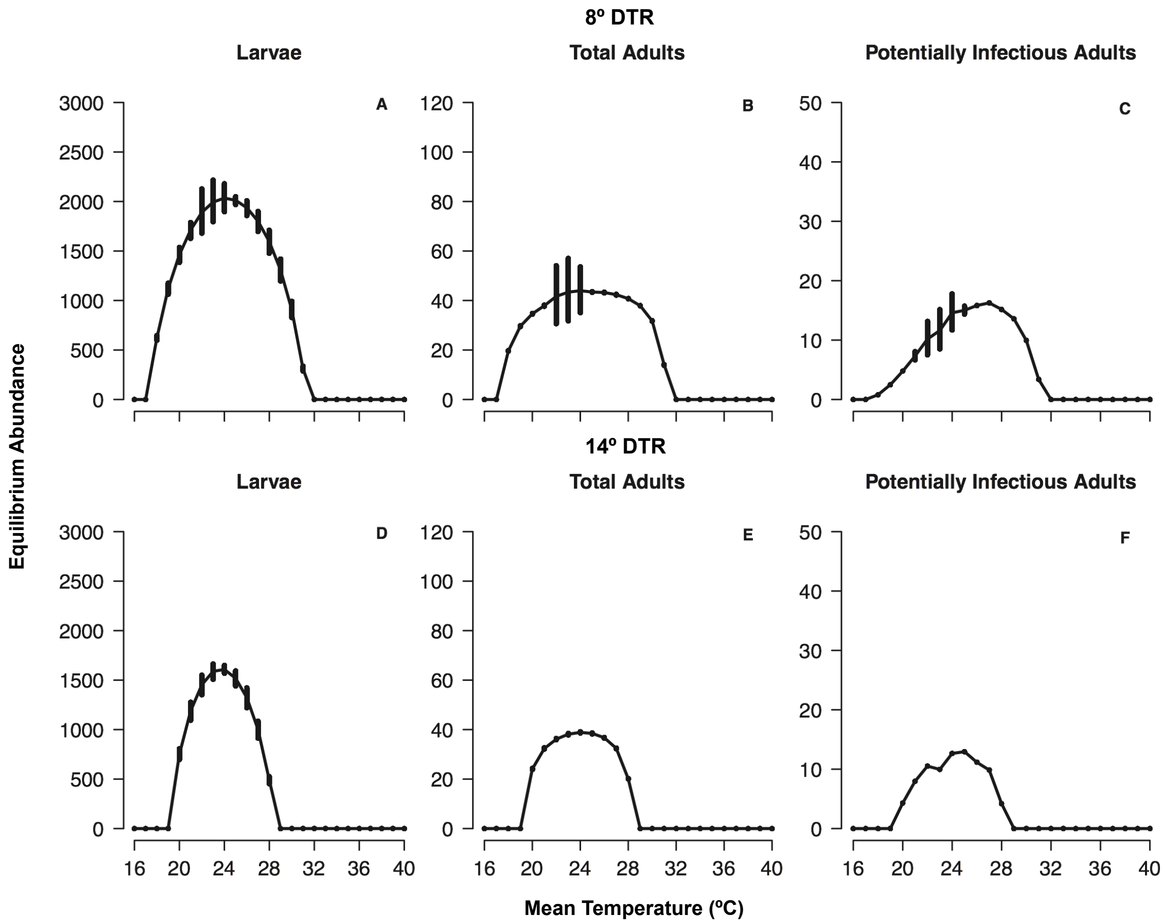

Figure S9: **Equilibrium abundance of model runs using a diurnal temperature driver.** The x-axis is the mean temperature around which the temperature is fluctuated. The vertical bars are the abundances (number of mosquitoes per liter of larval habitat) that the system cycles through at equilibrium. The line going through the bars is the average abundance. Panel A, B, and C are the larval, total adult and potentially infectious adult abundance with an 8°C diurnal temperature range, panel D, E, and F are the larval, total adult and potentially infectious adult abundance with a 14°C diurnal temperature range.

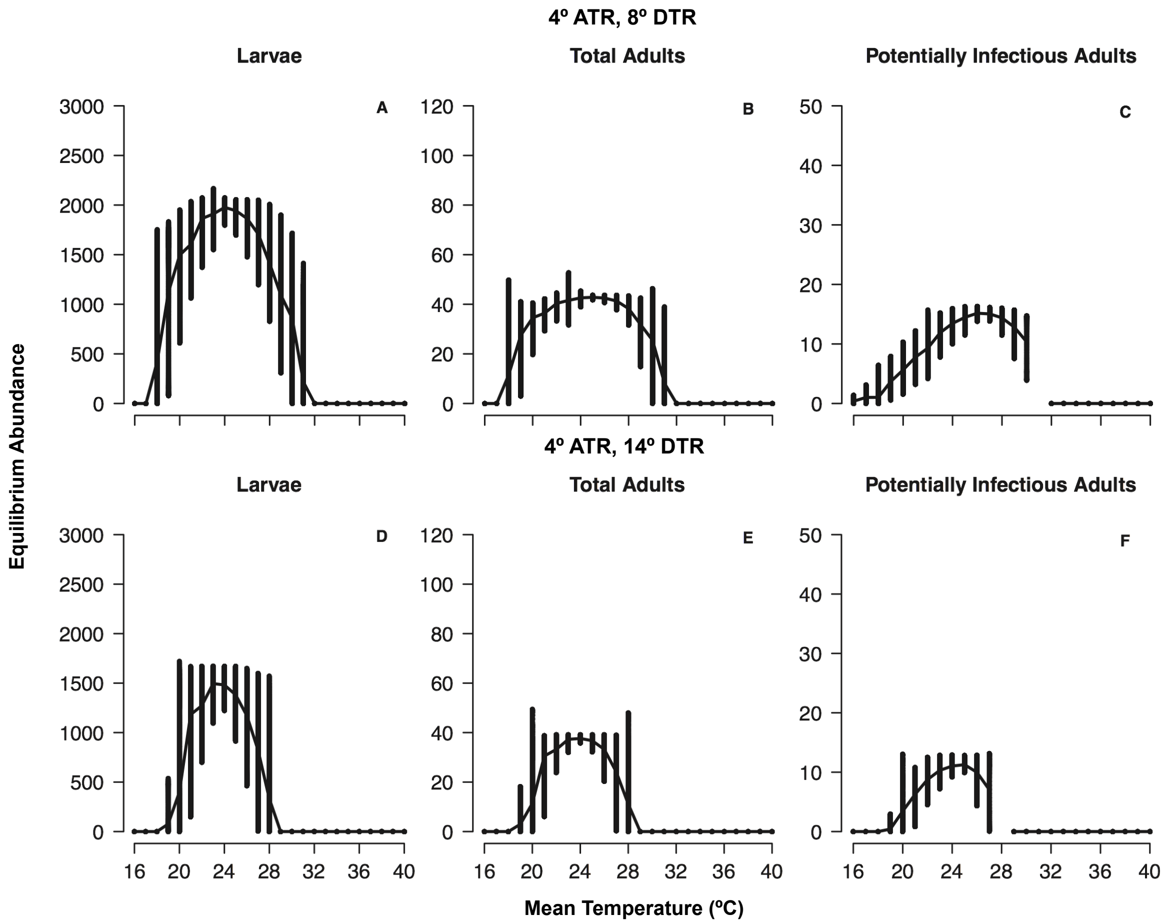

Figure S10: **Equilibrium abundance of model runs using a both an annual and diurnal temperature driver.** The x-axis is the mean temperature around which the temperature is fluctuated. The vertical bars are the abundances (number of mosquitoes per liter of larval habitat) that the system cycles through at equilibrium. The line going through the bars is the average abundance. All panels in this figure have a 4°C annual temperature range. Panel A, B, and C are the larval, total adult and potentially infectious adult abundance with an 8°C diurnal temperature range, panel D, E, and F are the larval, total adult and potentially infectious adult abundance with a 14°C diurnal temperature range. At some combinations of high temperature and large temperature fluctuations the solver was too stiff to handle the rapid changes in temperature and could not accurately capture the dynamics of the potentially infectious population.

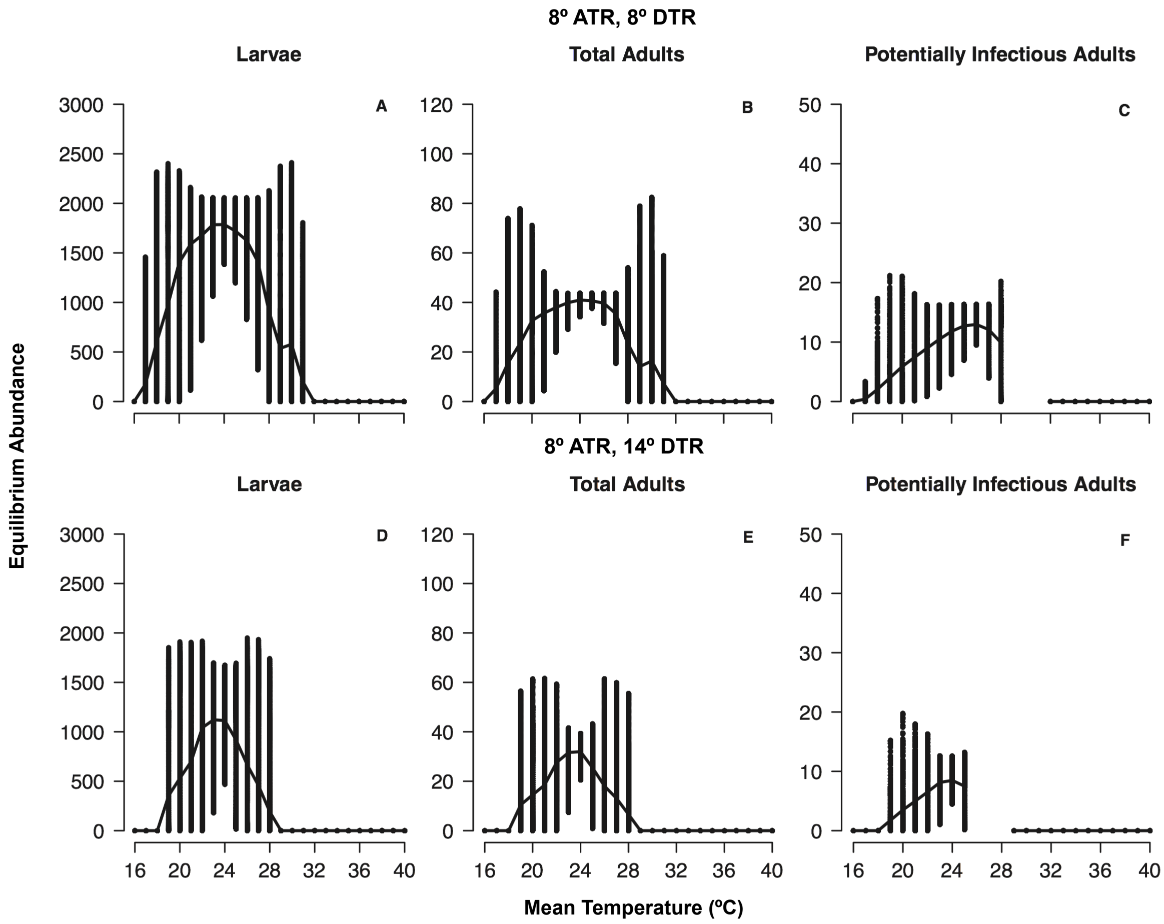

Figure S11: **Equilibrium abundance of model runs using a both an annual and diurnal temperature driver.** The x-axis is the mean temperature around which the temperature is fluctuated. The vertical bars are the abundances (number of mosquitoes per liter of larval habitat) that the system cycles through at equilibrium. The line going through the bars is the average abundance. All panels in this figure have a 8°C annual temperature range. Panel A, B, and C are the larval, total adult and potentially infectious adult abundance with an 8°C diurnal temperature range, panel D, E, and F are the larval, total adult and potentially infectious adult abundance with a 14°C diurnal temperature range. At some combinations of high temperature and large temperature fluctuations the solver was too stiff to handle the rapid changes in temperature and could not accurately capture the dynamics of the potentially infectious population.

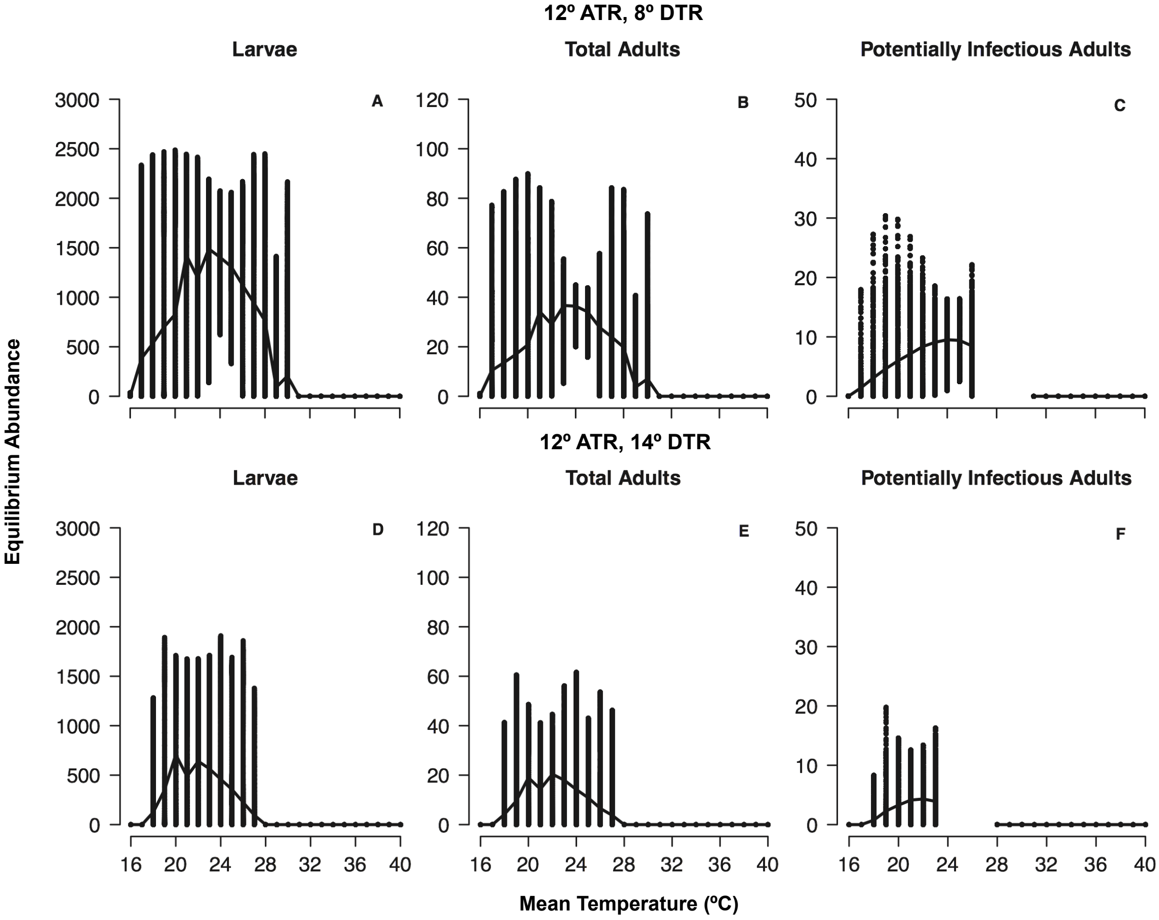

Figure S12: **Equilibrium abundance of model runs using a both an annual and diurnal temperature driver.** The x-axis is the mean temperature around which the temperature is fluctuated. The vertical bars are the abundances (number of mosquitoes per liter of larval habitat) that the system cycles through at equilibrium. The line going through the bars is the average abundance. All panels in this figure have a 12°C annual temperature range. Panel A, B, and C are the larval, total adult and potentially infectious adult abundance with an 8°C diurnal temperature range, panel D, E, and F are the larval, total adult and potentially infectious adult abundance with a 14°C diurnal temperature range. At some combinations of high temperature and large temperature fluctuations the solver was too stiff to handle the rapid changes in temperature and could not accurately capture the dynamics of the potentially infectious population.

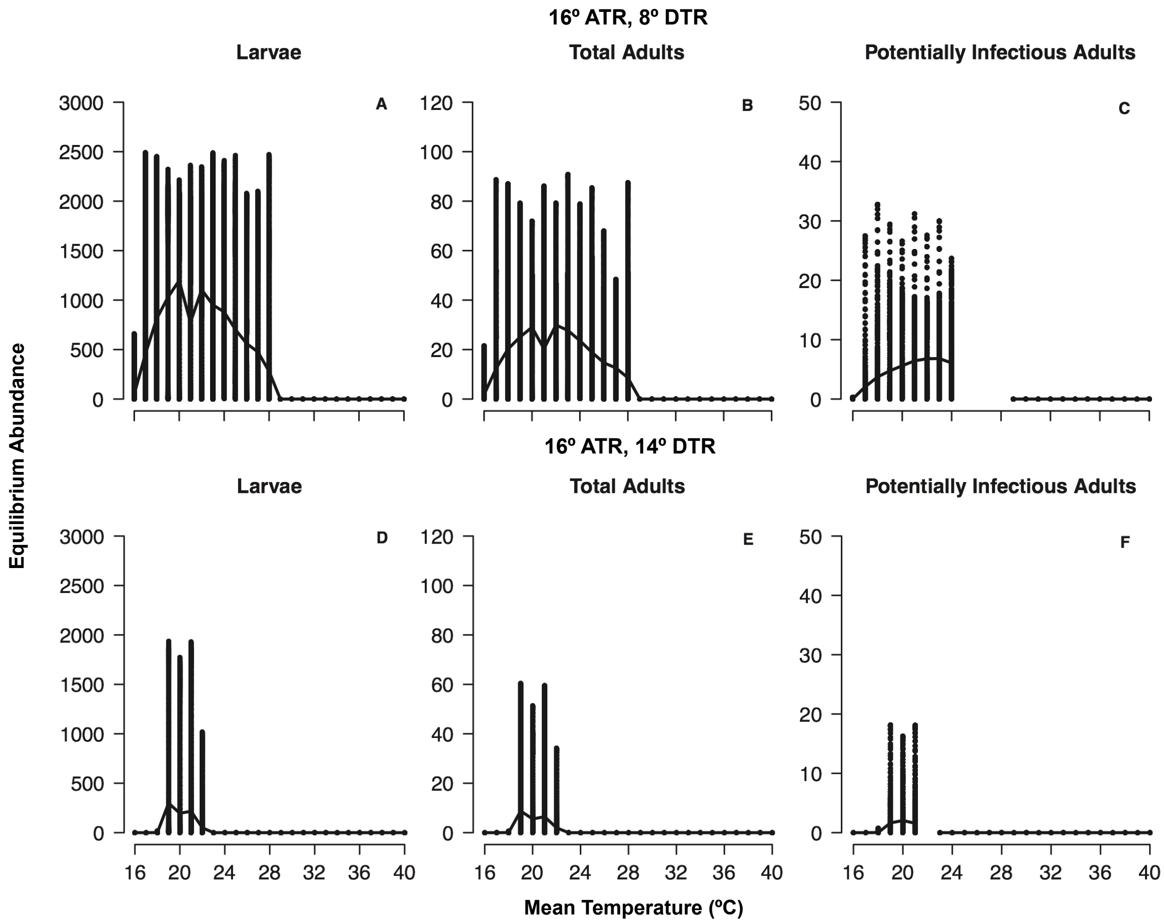

Figure S13: **Equilibrium abundance of model runs using a both an annual and diurnal temperature driver.** The x-axis is the mean temperature around which the temperature is fluctuated. The vertical bars are the abundances (number of mosquitoes per liter of larval habitat) that the system cycles through at equilibrium. The line going through the bars is the average abundance. All panels in this figure have a 16°C annual temperature range. Panel A, B, and C are the larval, total adult and potentially infectious adult abundance with an 8°C diurnal temperature range, panel D, E, and F are the larval, total adult and potentially infectious adult abundance with a 14°C diurnal temperature range. At some combinations of high temperature and large temperature fluctuations the solver was too stiff to handle the rapid changes in temperature and could not accurately capture the dynamics of the potentially infectious population.

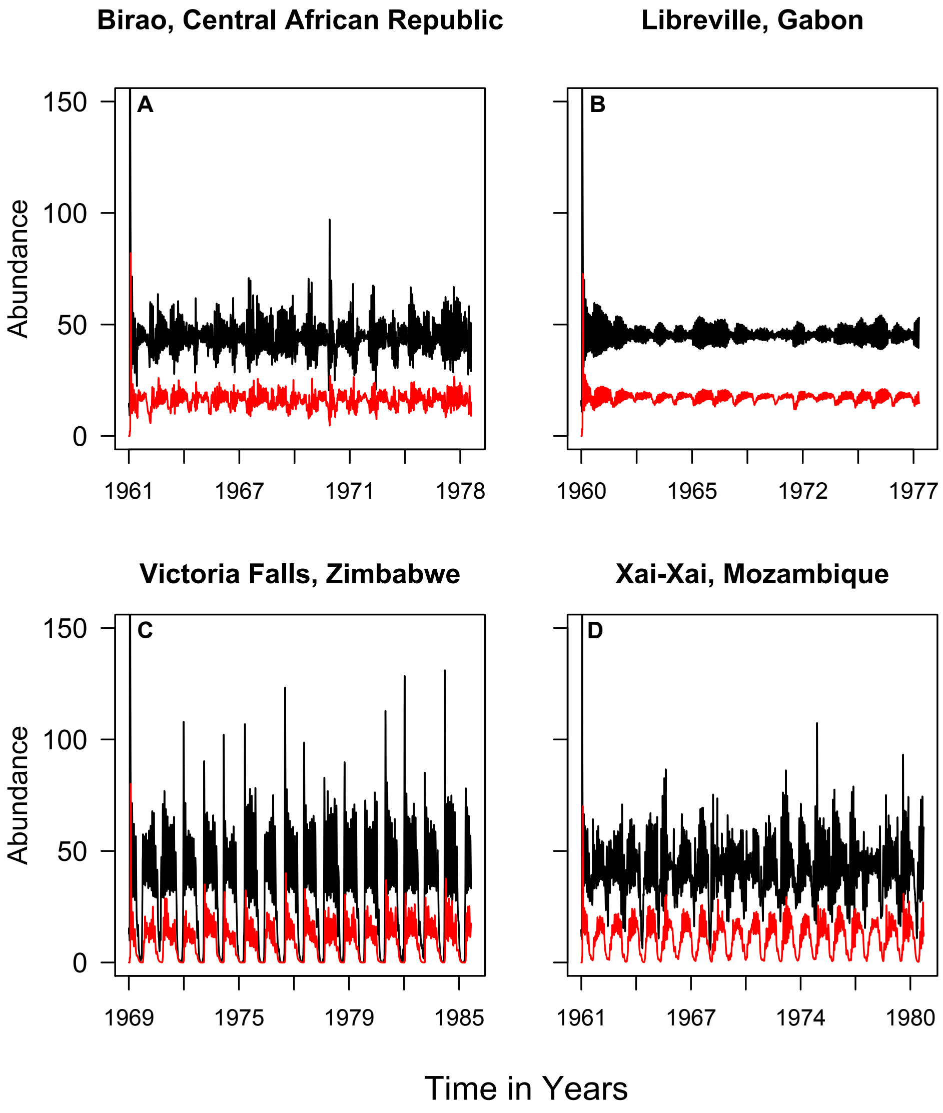

Figure S14: **Simulation results over the full time period for the four case study locations in Africa** The x-axis shows the year (note this axis differs between the plots), the y-axis shows the abundance. The black line shows the total adult population abundance and the red line shows the potentially infectious subset of the adult population. The abundance is number of mosquitoes per liter of larval habitat. Panel A) is Birao, Central African Republic, B) is Libreville, Gabon, C) is Victoria Falls, Zimbabwe, and D) is Xai-Xai, Mozambique.

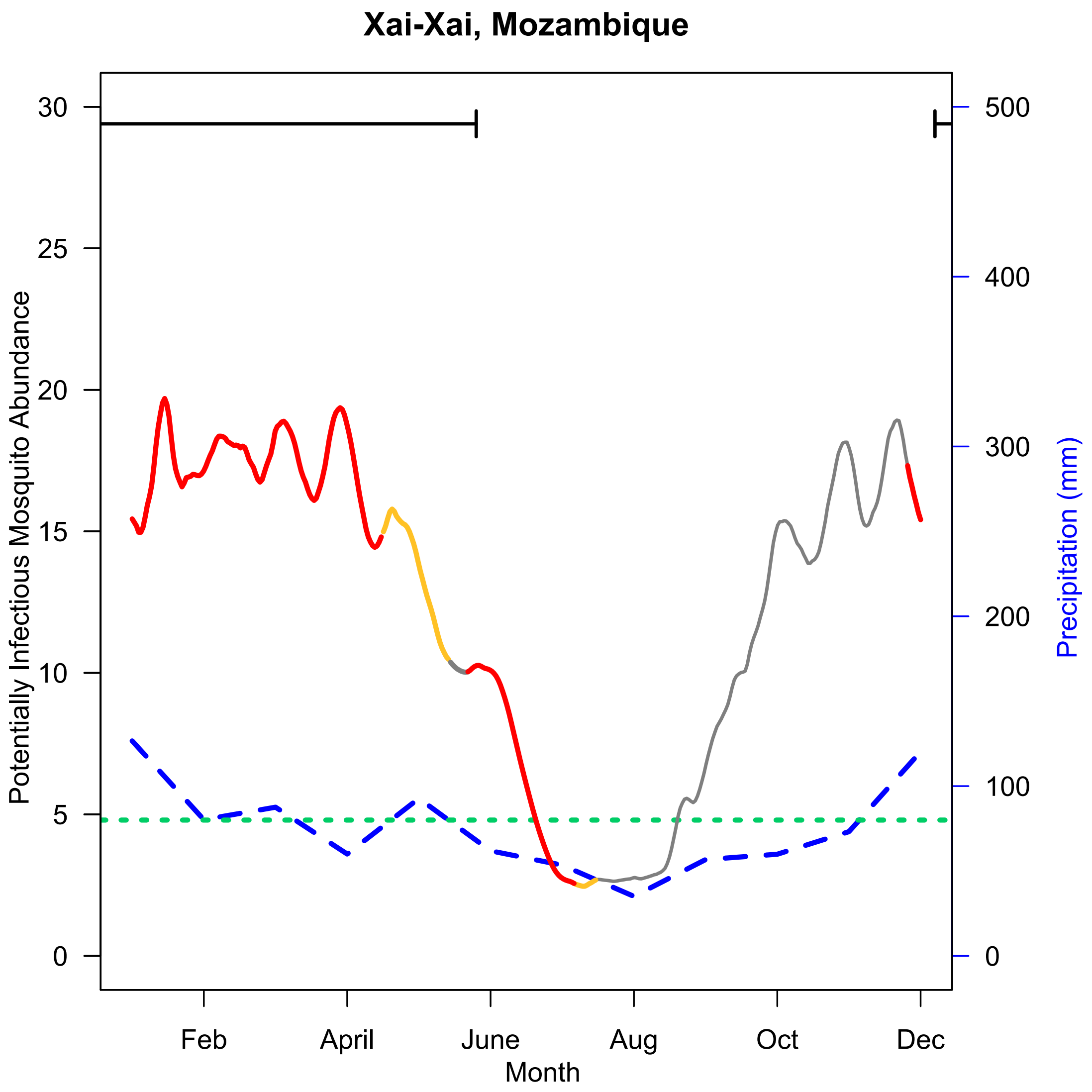

Figure S15: **Mean annual mosquito population dynamics, filtered by water availability.** This plot shows the model predictions for the potentially infectious mosquito population for Mozambique from 1980 –1990. The left axis corresponds to the mosquito population abundance (number of mosquitoes per liter of larval habitat) and the right y-axis corresponds with precipitation. The blue dashed line is the mean monthly precipitation and the horizontal green dotted line shows the 80mm precipitation threshold. The mosquito population line has been color coded to correspond with the availability of water. Recall that the model is not directly run with precipitation information, but is rather filtered once the temperature-dependent results are established. The red portions of the line correspond to populations in which the eggs were laid when precipitation was equal to or above the 80mm per month threshold. The orange portions of the mosquito population line shows the populations where precipitation has fallen below the threshold but mosquitoes that were already in the potentially infectious class are surviving. The grayed out portion of the line shows what the population would do if water was available year round. The black horizontal time index bars at the top of the panels mark the beginning and end of the malaria season.

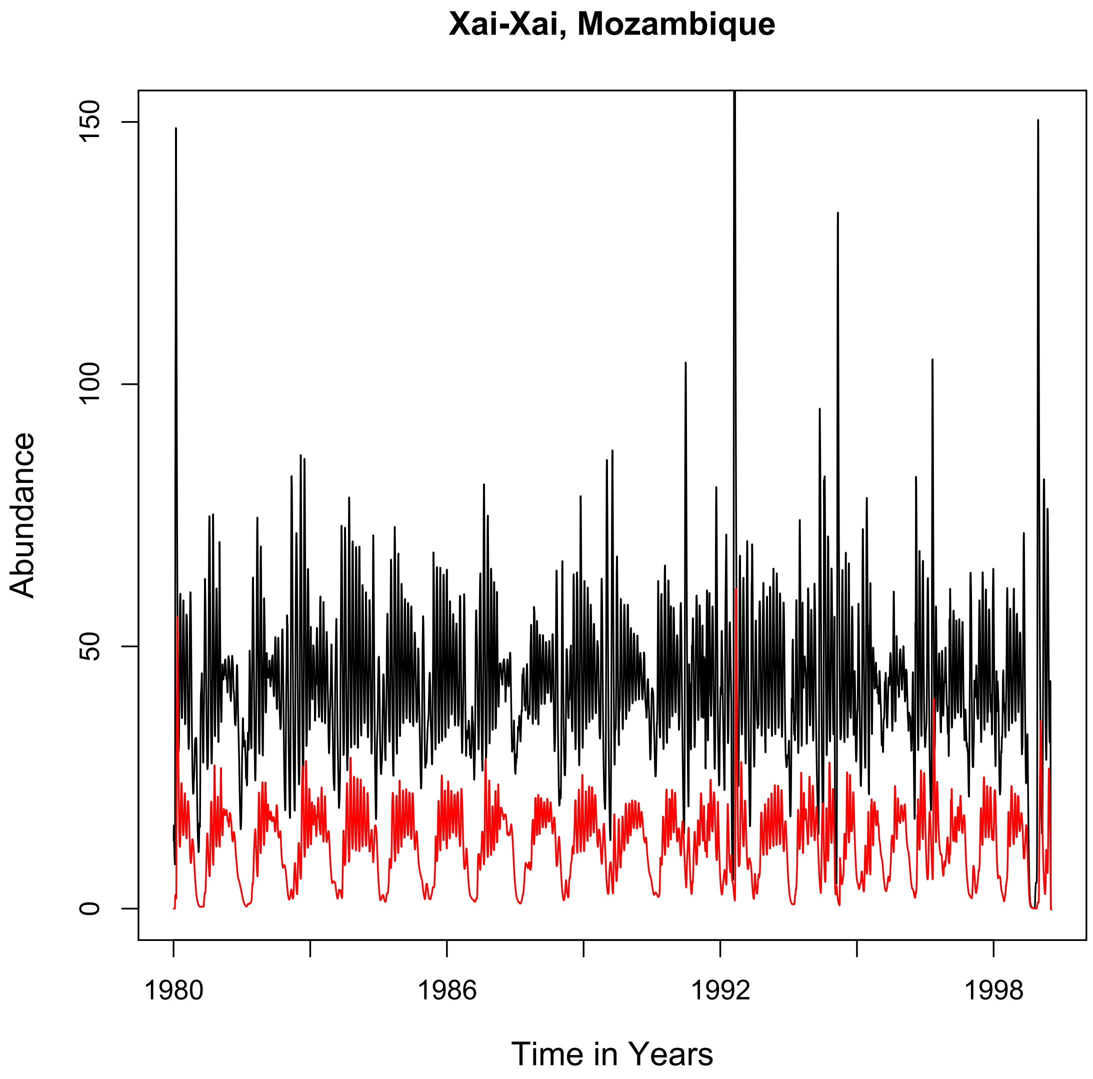

Figure S16: **Simulation results from Xai-Xai, Mozambique for the time period between 1980 –2000** The x-axis shows the year, the y-axis shows the abundance. The black line shows the total adult population and the red line shows the potentially infectious subset of the adult population. Note that the temperature data is much less complete after 1990 so the results from 1990 –2000 are not as robust as the first 10 years.

Table S1: Summary statistics for four African locations.

Location	Malaria Season (Months)	Mean Annual Precipitation (mm)	Precipitation > 80mm (months)	Mean Temp. (annual)	Annual Temp. Range
Birao, CAR	6	709.1	4	26.7	7.0
Libreville, Gab.	10	2881.7	9	25.9	2.8
Victoria Falls, Zim.	5	669.9	3	21.2	10.1
Xai-Xai, Moz.	6	1048.6	6	22.9	8.1

Table S2: Generalized linear model ranking results. The response variable is the malaria season (presence or absence).

Model	AICc	degrees of freedom
~Temperature-driven mosquito abundance + precipitation +location	43.37	42
~Precipitation + location	46.01	43
~Temperature-driven mosquito abundance +location	49.56	43
~1	67.88	47
