# Supplementary material for: The importance of temperature fluctuations in understanding mosquito population dynamics and malaria risk
Source: R Soc Open Sci. 2017 Mar 8;4(3):160969. doi: 10.1098/rsos.160969 (PMC5383843; doi:10.1098/rsos.160969)
Supplement: Code for Running Mosquito Population Model Simulations (Beck-Johnson et al. 2016) [file rsos160969supp2.pdf]

R code for model simulations for:

The importance of temperature fluctuations in understanding mosquito population dynamics and malaria risk

Lindsay M. Beck-Johnson[1,*], William A. Nelson[2], Krijn P. Paaijmans[3], Andrew F. Read[1,4,5], Matthew B. Thomas[2], Ottar N. Bjørnstad[1,4,5]

[1]Department of Biology, Center for Infectious Disease Dynamics, The Pennsylvania State University, University Park, PA, USA;
[2]Department of Biology, Queen's University, Kingston, Ontario, Canada;
[3]ISGlobal, Barcelona Ctr. Int. Health Res. (CRESIB), Hospital Clinic, Universitat de Barcelona;
[4]Department of Entomology, Center for Infectious Disease Dynamics, The Pennsylvania State University, University Park, PA, USA;
[5]Fogarty International Center, National Institutes of Health, Bethesda, MD, USA;
[*]L.Beck-Johnson@colostate.edu;
[*]Present address: Lindsay M. Beck-Johnson, Department of Biology, Colorado State University, Fort Collins, CO, USA

Overview of the Code

The simulation code for this model is divided into two parts. The first part runs the main simulation for the four main life stages, egg larva, pupa and adult. The second part runs the simulation to determine the number of mosquitoes that could potentially be infectious. The reason for this split is the first simulation is run on physiological (ϕ) time and the second simulation is run on normal time. It was not possible to run the second simulation on ϕ time because the temperature-dependent mosquito development rate function, which we use to determine the ϕ time, has a different functional form from the parasite development rate function. Therefore, it was not possible to transform the parasite development rate using the ϕ scale transformation (for the details of the main model transformation to the ϕ scale please see [1]; for details of the parasite development rate see the methods section of the main text).

Main Model Simulation Code

The following code runs the main simulation for the four main mosquito life stages. Encompassed in this model is all the information required to determine the adult age structure and the potentially infectious adult mosquitoes except for the parasite development rate which will be determined in the next section of code.

```
> library(PBSddesolve) #this package is required for both this
> #simulation and the simulation for the potentially infectious
> #mosquitoes
>
```

```
> mosquito_model=function(t,y,parms){
+ #The following commands define the model parameters and the y
+ #vector, which defines the state variables. The objects in the
+ #y vector will be tracked throughout and can be called
+ #by pastvalues. The parameters are defined in
+ #Beck-Johnson et al. 2013.
+
+ rho=parms[1]
+ gammaE=parms[2]
+ gammaL=parms[3]
+ gammaP=parms[4]
+ alphaI=parms[5]
+ sigma.exp=parms[6]
+ A0=parms[7]
+ L0=parms[8]
+ P0=parms[9]
+ E0=parms[10]
+ mu0=parms[11]
+ mu1=parms[12]
+ mu2=parms[13]
+ mu3=parms[14]
+ mu4=parms[15]
+ mu5=parms[16]
+ TEMP=parms[17]
+
+
+ A=y[1]#adult state variable
+ L=y[2]#larval state variable
+ SE=y[3]#egg survivorship
+ SL=y[4]#larval survivorship
+ SP=y[5]#pupal survivorship
+ SA=y[6]#adult survivorship
+ tt=y[7]#this is time on the transformed scale
+ RA=y[8]
+
+ M=m(tt,TEMP); #M is temperature. This line of code calls the m
+ #function which tracks temperature. Throughout the code M^1.726 is
+ #the conversion to the phi scale
+
+
+
+ ##pastvalues
+
+ #This is the section of the code that allows the model to
+ #properly track the delays.
```

```
+ #The delays (lambda1--lambda6) set the length of the time delays,
+ #remember that the delays become fixed on the phi time scale.
+ #The lags section tells the simulation to keep track of the
+ #current simulation time (t) (remember it is on the phi scale)
+ #minus each of the delays. The if statements are necessary
+ #because the model must be past the time length of the delay
+ #before it will properly keep track of pastvalues.
+
+ #delays
+ lambda1=1/(gammaE*alphaI) #delay through egg
+ lambda2=1/(gammaL*alphaI) #delay through larva
+ lambda3=1/(gammaP*alphaI) #delay through pupa
+ lambda4=(lambda1+lambda2) #cumulative delay from egg
+ # through larva
+ lambda5=(lambda2+lambda3) #cumulative delay from larva
+ # through pupa
+ lambda6=(lambda1+lambda2+lambda3) #cumulative delay from egg
+ #through pupa (time to adult)
+
+ #lags
+ if(t>lambda1){lag1=pastvalue(t-lambda1)}
+ if(t>lambda2){lag2=pastvalue(t-lambda2)}
+ if(t>lambda3){lag3=pastvalue(t-lambda3)}
+ if(t>lambda4){lag4=pastvalue(t-lambda4)}
+ if(t>lambda5){lag5=pastvalue(t-lambda5)}
+ if(t>lambda6){lag6=pastvalue(t-lambda6)}
+
+ #-------------------------------------------------------------
+
+ #The following two equations are the mortality parts of the adult
+ #and larval state equations. These do not have delays in them.
+ dAdt=-((mu0/(M^1.726))*exp(((M-mu1)/mu2)^4))*A #mortality in adults
+ dLdt=-(((gammaL*mu3)/(M^1.726))*
+ exp(((M-mu4)/mu5)^(2)+(sigma.exp*L)))*L
+ #mortality in larvae
+
+ #Cohort survivorship. These equations are built on using if
+ #statements so that all the delays are properly accounted for.
+ #egg stage survivorship
+ S1=exp(-SE);if(t>lambda1){S1=exp(lag1[3]-SE)}
+ #egg-larval stage survivorship
+ S2=exp(-SL);if(t>lambda2){S2=exp(-lag2[3]+lag2[4]-SL)};
+ if(t>lambda4){S2=exp(lag4[3]-lag2[3]+lag2[4]-SL)}
+  #egg-pupal stage survivorship
+ S3=exp(-SP);if(t>lambda3){S3=exp(-lag3[4]+lag3[5]-SP)};
```

```
+ if(t>lambda5){S3=exp(-lag5[3]+lag5[4]-lag3[4]+lag3[5]-SP)};
+ if(t>lambda6){S3=exp(lag6[3]-lag5[3]+lag5[4]-lag3[4]+lag3[5]-SP)}
+ #-------------------------------------------------------------------
+
+
+ #recruitment from birth
+
+ #eggs recruit into L, not out
+ if(t>lambda1){dLdt=dLdt+rho*lag1[1]*S1}
+ #larvae can mature out of L into P
+ if(t>lambda4){dLdt=dLdt-rho*lag4[1]*S2}
+ #pupae recruit into adult stage
+ if(t>lambda6){dAdt=dAdt+rho*lag6[1]*S3}
+
+ #recruitment of inoculated individuals. Inoculated individuals
+ #are those present at the beginning of the simulation, and
+ #their fate must be handled separately from later individuals,
+ # whose history can be tracked via pastvalues. The initial
+ #inoculated individuals are uniformly distributed through
+ #their respective stages.
+
+ #following inoculated individuals through the juvenile stages
+ if(t<=lambda2){dLdt=dLdt-(L0/lambda2)*S2} #larvae out
+ if(t<=lambda1){dLdt=dLdt+(E0/lambda1)*S1}  #eggs in
+ if(t>lambda2 && t<=lambda4){dLdt=dLdt-(E0/lambda1)*S2} #eggs out
+
+ #follow the inoculated individuals through the adult stage
+ if(t<=lambda3){dAdt=dAdt+(P0/lambda3)*S3} #pupae in
+ if(t>lambda3 && t<=lambda5){dAdt=dAdt+(L0/lambda2)*S3} #larvae in
+ if(t>lambda5 && t<=lambda6){dAdt=dAdt+(E0/lambda1)*S3} #eggs in
+
+ #------------------------------------------------------------------
+
+ #mortality functions
+
+ dSE=(gammaE*mu3/(M^1.726))*exp(((M-mu4)/mu5)^2)
+
+  #exponential density dependence
+ dSL=((gammaL*mu3/(M^1.726))*exp(((M-mu4)/mu5)^(2)+(sigma.exp*L)))
+ #linear density dependence
+ #dSL=((gammaL*mu3/(M^1.726))*exp(((M-mu4)/mu5)^2)+(sigma.lin*L)/
+ #(M^1.726))
+
+ dSP=(gammaP*mu3/(M^1.726))*exp(((M-mu4)/mu5)^2)
+ dSA=(mu0/(M^1.726))*exp(((M-mu1)/mu2)^4)
```

```
+
+ #follow how time changes on the phi scale with dt/dphi
+ dtdphi=1/(M^1.726)
+
+ #Follow the flux (recruitment) into the Adult class.
+ #The first 3 equations follow the inoculated individuals.
+ if(t<=lambda3){RA=(P0/lambda3)*(S3)}
+ if(t>lambda3 && t<=lambda5){RA=(L0/lambda2)*(S3)}
+ if(t>lambda5 && t<=lambda6){RA=(E0/lambda1)*(S3)}
+ if(t>lambda6){RA=rho*lag6[1]*(S3)}
+
+ return(c(dAdt,dLdt,dSE,dSL,dSP,dSA,dtdphi,RA)) #returns adult
+ #& larval abundance, egg, larval, pupal and adult survivorship,
+ #time (actual time not phi time) and adult recruitment
+
+ }#end of mosquito_model
> #parameters
> rho=0.156 #Birth Rate, 150 eggs/female
> gammaE=6  #proportion of time spent in egg stage
> gammaL=3/2 #proportion of time spent in larval stage
> gammaP=6 #proportion of time spent in pupal stage
> alphaI=2.87e-4#devel scalar for immature development
> sigma.exp=0.001326052 #den. dependent param for exponential model
> sigma.lin=3.687853e-5 #den. dependent param for linear model
> A0=10 #initial value of Adults
> L0=10 #initial value of Larvae
> P0=10 #initial value of Pupae
> E0=10 #initial value of Eggs
> mu0=0.08858675 #mu0-mu2 are the adult mortality parameters
> mu1=21.21144138
> mu2=14.85178065
> mu3=0.02 #mu3-mu5 are the larval mortality parameters
> mu4=23
> mu5=6.5
> #The TEMP parameter and the m function determine the temperature
> #driver when running the model at constant temperatures or using a
> #fluctuating sine function.
>
> # If TEMP is equal to -1 the temperature function will fluctuate.
> #If TEMP is set to a number greater than 0 it will be constant.
> #The code below shows a constant temperature of 28 and
> #the examples of fluctuating temperature function all
> #fluctuating around a mean of 28.
>
> TEMP=-1
```

```
> #TEMP=28
>
> #Several temperature functions were used for the exploration of
> #temperature fluctuations (see the main text for details). Below is
> #an example of each.
>
> #Seasonal fluctuation only (4 (+/-2) degrees in this example)
> m=function(tt,TEMP){
+ if(TEMP==-1){return((28+2*sin(2*pi*(tt-125.7)/365)))};
+ if(TEMP>0){return(TEMP)}
+ } # end m--temp function
> #Diurnal fluctuation only (8 (+/-4) degrees in this example)
> m=function(tt,TEMP){
+ if(TEMP==-1){return((28+4*sin(2*pi*(tt-(14/24))/1)))};
+  if(TEMP>0){return(TEMP)}
+ } # end m--temp function
> #Diurnal and seasonal fluctuation
> #(diurnal 8 degree and seasonal 4 degree in this example)
> m=function(tt,TEMP){
+ if(TEMP==-1){return((28+2*sin(2*pi*(tt-125.7)/365))+
+ 4*sin(2*pi*(tt-(14/24))/1))};
+ if(TEMP>0){return(TEMP)}
+ } # end m--temp function
> #parameter vector
> parms=c(rho,gammaE,gammaL,gammaP,alphaI,sigma.exp,A0,L0,
+ P0,E0,mu0,mu1,mu2,mu3,mu4,mu5,TEMP)
>
> #y.out=dde(y=c(A0,L0,0,0,0,0,0,0),times=seq(0,1.5e6,10),
> #func=model_fluc,parms=parms,tol=1e-8,hbsize=100000)
> #this will run the model.
> #times determines the length of time it will run.
> #Here y sets the initial conditions for the state variables
>
```

Potentially Infectious Mosquito Simulation Code

This simulation takes the information about the temperature, the recruitment into the adult stage and the timing from the main model simulation and uses them to calculate the number of adult mosquitoes in any given cohort that could survive longer than the predicted parasite extrinsic incubation period (EIP). In order for this code to work the main model simulation must be run first. This function will use the same temperature function (*m*) as the main simulation.

```
> #the following code gives correct timing of the recruitment.
> #y.out[,8] calls the time from the previous simulation output
```

```
> #y.out[,9] calls the adult recruitment from the main model
> #simulation output
> #flux=diff(y.out[,9])/diff(y.out[,8])
>
> #the following code gives the number of recruits
> #at each point in time
> #recruit=approxfun(y.out[-dim(y.out)[1],8],flux)
>
>
> #This model is structured the same way as the main
> #simulation model code above.
>
> pim_model=function(t,y,parms){
+ D=parms[1]#dscale
+ K1=parms[2]
+ L1=parms[3]
+ K2=parms[4]
+ L2=parms[5]
+ TEMP=parms[6]
+ mu0=parms[7]
+ mu1=parms[8]
+ mu2=parms[9]
+
+ W=y[1]
+ tauA=y[2]
+ S=y[3]
+ S.pi=y[4]
+ M=y[5]
+ PI=y[6]
+
+ #We track current temperature and past temperature
+ #using the following lines of code
+ M=m(t,TEMP) #current temperature
+ MM=m(t-tauA,TEMP) #past temperature
+
+ #pastvalue
+ #The only delay in this model is the length of the
+ #parasite EIP which is tauA. Unlike the delays
+ #in the main model this delay is variable
+ #through time in the simulation
+ #for this past value we are only interested
+ #in tracking the delay of the adult
+ #survivorship S which is the 3rd
+ #element of y
+ if(W>1){S.lag=pastvalue(t-tauA)[3]}
```

```
+
+ #development rate function of the parasite at
+ #the current time (ha) and in the past (hap)
+ ha=D*(exp(-(M/L2)^K2)-exp(-(M/L1)^K1-(M/L2)^K2))
+ if(W>1) hap=D*(exp(-(MM/L2)^K2)-exp(-(MM/L1)^K1-(MM/L2)^K2))
+
+ dW=ha
+
+ dtauA=1; if(W>1){dtauA=1-ha/hap}
+
+ dSdt=(mu0)*exp(((M-mu1)/mu2)^4) #adult survival
+
+ S.pi=exp(-S);if(W>1){S.pi=exp(-S+S.lag)} #survival
+ # to potentially infectious stage
+
+ #The following line of code tracks the potentially
+ #infectious mosquitoes. These are initially 0
+ if(W>1){PI=recruit(t-tauA)*S.pi}
+
+ return(c(dW,dtauA,dSdt,S.pi,M,PI))
+ #returns the EIP development rate, the length of the delay
+ #adult survivorship, survivorship to PI stage
+ #the temperature and the potentially infectious cumulative
+ #abundance
+
+ }#end pim_model
> #parameters
> mu0=0.08858675 #mu0-mu2 are the same adult
> #mortality parameters as in the main model
> mu1=21.21144138
> mu2=14.85178065
> #the following parameters are all used in the
> #EIP development function
> D=0.1071171
> K1=6.3436511
> L1=22.9641799
> K2=48.6648879
> L2=34.5885507
> #parameter vector
> parms1=c(D,K1,L1,K2,L2,TEMP,mu0,mu1,mu2)
>
> #y.outpim=dde(y=c(0,0,0,0,0,0),times=seq(0,50000,1),
> #func=pim_model,parms=parms1,tol=1e-10,hbsize=1000000)
> #this line of code will run this model.
> #Remember the main simulation must be run first.
```

```
> #Note: this simulation will not return numbers for the potentially
> # infectious mosquito abundance for times past those run in
> #the main model, so the maximum time must be chosen appropriately.
```

Code to Run GLM

The following code requires the use of the Supplemental File "seasonality.csv". This csv file contains binomial indicators for months of malaria season, and precipitation above the 80mm threshold for all four locations. Additionally there is an average number of potentially infectious mosquitoes (per liter of breeding habitat) by month for each location.

```
> require(AICcmodavg)
> dat<-read.csv("seasonality.csv",header=T)
> head(dat)

  month Location mal.seas mosquito precp
1     1    Birao        0 13.48294     0
2     2    Birao        0 15.81454     0
3     3    Birao        0 17.05987     0
4     4    Birao        0 14.66199     0
5     5    Birao        0 16.25432     0
6     6    Birao        1 18.02920     1

> #mosquito abudance+location model
> fit1=glm(mal.seas~mosquito+as.factor(Location),
+ data=dat,family=binomial())
>
> #precpitiation+location model
> fit2=glm(mal.seas~precp+as.factor(Location),
+ data=dat,family=binomial())
>
> #mosquito abundance+precipitation+location model
> fit3=glm(mal.seas~mosquito+precp+as.factor(Location),
+ data=dat,family=binomial())
>
> fit4=glm(mal.seas~1, data=dat,family=binomial())
>
> AICc(fit1)

[1] 49.56231

> AICc(fit2)

[1] 46.00567

> AICc(fit3)
```

```
[1] 43.372

> AICc(fit4)

[1] 67.87712
```

References

[1] Beck-Johnson, L. M., Nelson, W. A., Paaijmans, K. P., Read, A. F., Thomas, M. B. & Bjornstad, O. N. 2013 The effect of temperature on anopheles mosquito population dynamics and the potential for malaria transmission. *Plos One* **8**, e79276.
